# Neurogenin2 Directs Granule Neuroblast Production and Amplification while NeuroD1 Specifies Neuronal Fate during Hippocampal Neurogenesis

**DOI:** 10.1371/journal.pone.0004779

**Published:** 2009-03-10

**Authors:** Laurent Roybon, Tord Hjalt, Simon Stott, Francois Guillemot, Jia-Yi Li, Patrik Brundin

**Affiliations:** 1 Neuronal Survival Unit, Department of Experimental Medical Science, Wallenberg Neuroscience Center, Lund, Sweden; 2 Division of Developmental Neurobiology, MRC National Institute for Medical Research, The Ridgeway, London, United Kingdom; 3 Division of Molecular Neurobiology, MRC National Institute for Medical Research, The Ridgeway, London, United Kingdom; University of Washington, United States of America

## Abstract

The specification and differentiation of dentate gyrus granule neurons in the hippocampus require temporally and spatially coordinated actions of both intrinsic and extrinsic molecules. The basic helix-loop-helix transcription factor Neurogenin2 (Ngn2) and NeuroD1 are key regulators in these processes. Based on existing classification, we analyzed the molecular events occurring during hippocampal neurogenesis, primarily focusing on juvenile animals. We found that Ngn2 is transiently expressed by late type-2a amplifying progenitors. The Ngn2 progenies mature into hippocampal granule neurons. Interestingly, the loss of Ngn2 at early stages of development leads to a robust reduction in neurogenesis, but does not disturb granule neuron maturation *per se*. We found that the role of Ngn2 is to maintain progenitors in an undifferentiated state, allowing them to amplify prior to their maturation into granule neurons upon NeuroD1 induction. When we overexpressed Ngn2 and NeuroD1 *in vivo*, we found NeuroD1 to exhibit a more pronounced neuron-inductive effect, leading to granule neuron commitment, than that displayed by Ngn2. Finally, we observed that all markers expressed during the transcriptional control of hippocampal neurogenesis in rodents are also present in the human hippocampus. Taken together, we demonstrate a critical role of for Ngn2 and NeuroD1 in controlling neuronal commitment and hippocampal granule neuroblast formation, both during embryonic development and in post-natal hippocampal granule neurogenesis.

## Introduction

Neurons are born not only during development of the central nervous system, but neurogenesis also continues into adulthood. In both rodent and human adult brain, neurogenesis is active in two distinct zones of the forebrain: the subventricular zone (SVZ) of the lateral ventricle and the subgranular zone (SGZ) of the hippocampal dentate gyrus (DG) [1]–[5]. The molecular mechanism underlying neurogenesis in the DG is not fully understood. Clearly, a cascade of transcriptional events controls the specification of neuronal identity in the DG [3], [6]–[8], but details of the expression pattern and function of each transcription factor remain elusive.

The paired-box homeodomain transcription factor Pax6 and the bHLH transcription factors Ngn2 and NeuroD1 are important when cells acquire a pan-neuronal character and a specific neuronal subtype [9], [10]. In the developing neocortex, Pax6 is expressed in dividing radial glial cells at the ventricular surface [9]. In the adult hippocampus, Pax6 is expressed in astrocytes in the SGZ and are considered to be the true stem cells [9], [11]. Loss- and gain-of-function studies indicate that Pax6 is involved in regulating the proliferation of neocortical and hippocampal progenitors [9], [12]–[16]. During neocorticogenesis, high concentrations of Pax6 induce the expression of the bHLH transcription factor Neurogenin2 (Ngn2) [17]. In turn, Ngn2 causes cell cycle exit [18], an event that takes place when NeuroD1 starts to be expressed [19]. In the developing hippocampus, the absence of Ngn2 leads to a reduced number of granule neurons [20]. NeuroD1, on the other hand, is essential for the differentiation and survival of hippocampal granule neurons [10]. Interestingly, in the absence of Ngn2, NeuroD1 can still be activated and neuronal differentiation can still take place [20]. This has led to the idea that the primary role of Ngn2 is not to direct neuronal differentiation.

Only a small number of studies have addressed the role of Ngn2 and NeuroD1 in hippocampal neurogenesis [10], [20], [21]. Therefore, we now re-examine their roles in hippocampal neurogenesis in detail, using *gain*- and *loss-of-function* experiments. We first establish a hierarchy of transcriptional events that occur during neurogenesis in the DG and then define the place that Ngn2, NeuroD1 and other transcription factors have in this cascade. We find that a lack of Ngn2 expression result in a markedly smaller hippocampus and an almost complete absence of the DG. We show that Ngn2 is required for granule neuroblasts production/amplification. *Gain-of-function* of NeuroD1 during development of the DG results in an efficient generation of granule neuroblasts, an effect that we do not observe when we overexpress Ngn2. Finally, we demonstrate that the same transcription factors and cellular markers seen in mouse and rat tissue are also present in the human hippocampus.

## Results

### Expression of transcription factors and cellular markers define different phases of postnatal hippocampal granule neuron maturation

**Figure 1:**
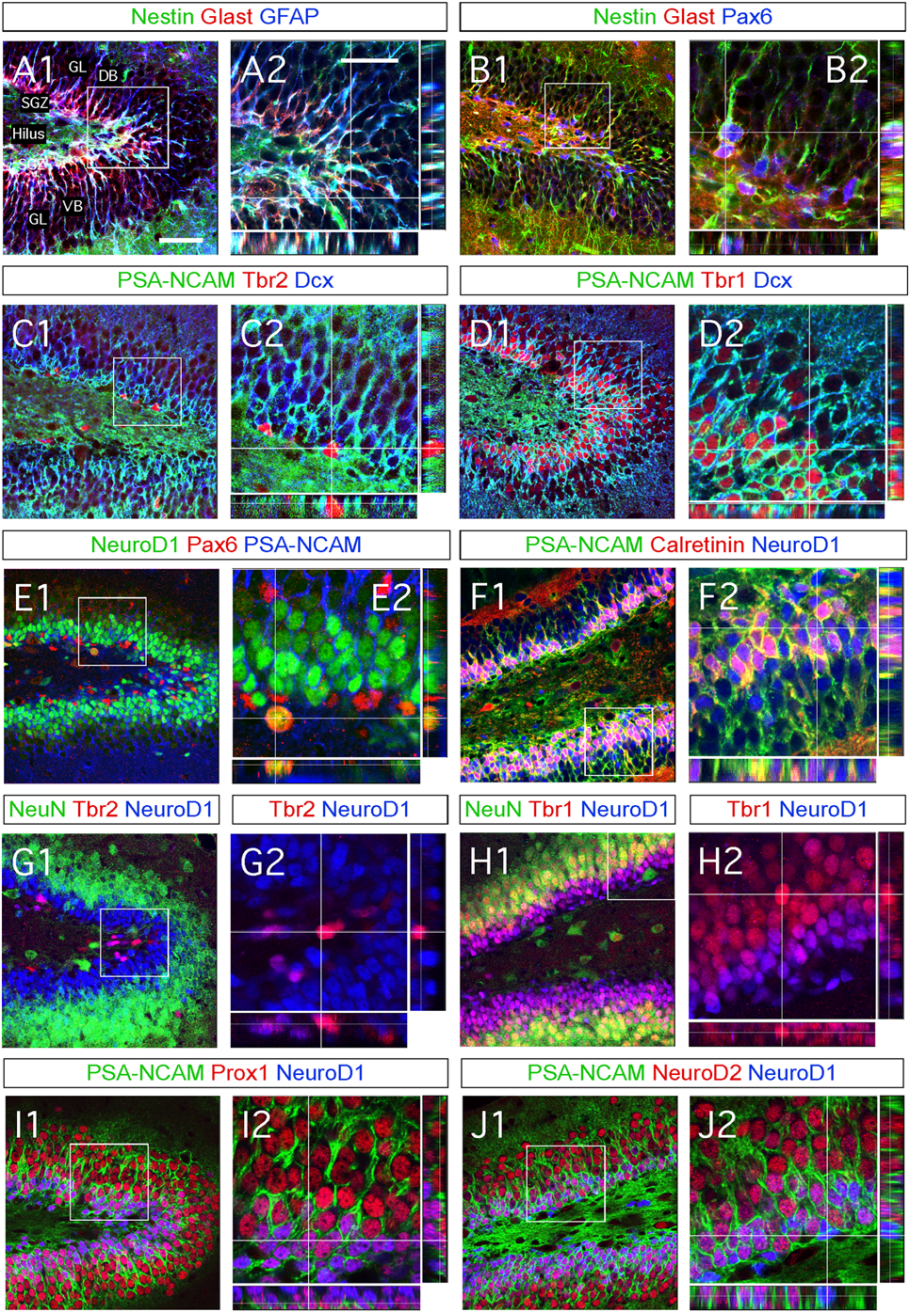
Characterization of the molecular signatures defining each phases of cell maturation during hippocampal granule neuronal differentiation, in two-weeks old newborn mice. (A1–J2) Indirect immunofluorescence performed on hippocampal coronal sections from 2 weeks old newborn mice. (A1–B2) Type-1 radial glia stem cells are identified by the co-expression of Nestin, Glast, GFAP and Pax6. Radial glia cells locate in both dorsal and ventral blades of the DG. (C1–C2) Type-2 amplifying progenitors expressing Tbr2 rarely co-express PSA-NCAM or Dcx. (D1–D2) Type-3 maturing granule neurons co-express Tbr1, PSA-NCAM and Dcx. (E1–E2) Type-3 maturing granule neurons co-express NeuroD1 but not Pax6. Pax6 progenitors co-expressing NeuroD1 were rarely found and located in the hilus. (F1–F2) Granule neurons maturation occurs through the expression of NeuroD1, PSA-NCAM and Calretinin. (G1–G2) The transition from type-2 amplifying progenitors to mature neurons takes place when the type 2 cells express Tbr2 followed by expression of NeuroD1 and thereafter NeuN. (H1–H2) The transition from type-3 amplifying progenitors to mature granule neurons occurs through the expression of NeuroD1, Tbr1 and NeuN. (I1–I2) The expression of Prox1 starts soon after that of NeuroD1. (J1–J2) NeuroD2 expression starts soon after that of NeuroD1 and persists in mature granule neurons, in contrast to NeuroD1. Panels A2–J2 represent a high magnification of framed areas in corresponding panels A1–J1. Rectangular images on the bottom and right of the panels A2–J2 represent projected images of 14-Z stacks (total of 10–14 µm thick). The white crosshairs in these panels were positioned to show co-expression of markers of interest in single cells. See each panel label. SGZ = subgranular zone, GL = granule layer, DB = dorsal blade and VB = ventral blade. Scale bars: 50 µm (A1, B1, C1, D1, E1, F1, G1, H1, I1 and J1), 25 µm (A2, B2, C2, D2, E2, F2, G2, H2, I2 and J2).

We first analyzed in detail the chronology of expression of different transcription factors and cell-specific markers during DG granule neuron formation and correlated our findings to the previously established classification of adult hippocampal neurogenesis [3]. The current classification describes hippocampal stem cells and progenies into three categories (type-1, -2 and -3 cells) depending on the markers they express as they mature. In the adult mouse DG, only a very small proportion of cells undergo mitosis at any one given time [22]. Consequently, in adult animals it is difficult to analyze which transcription factors are involved in the transition phase from one cell category to the next. Therefore, we studied 2 week-old rodents which have higher numbers of maturing granule neurons in the DG. We performed triple immunohistochemistry for all markers of interest and compared results from 2 week- and 2 month-old mice (Figure 1 and figure S1).

**Figure 2:**
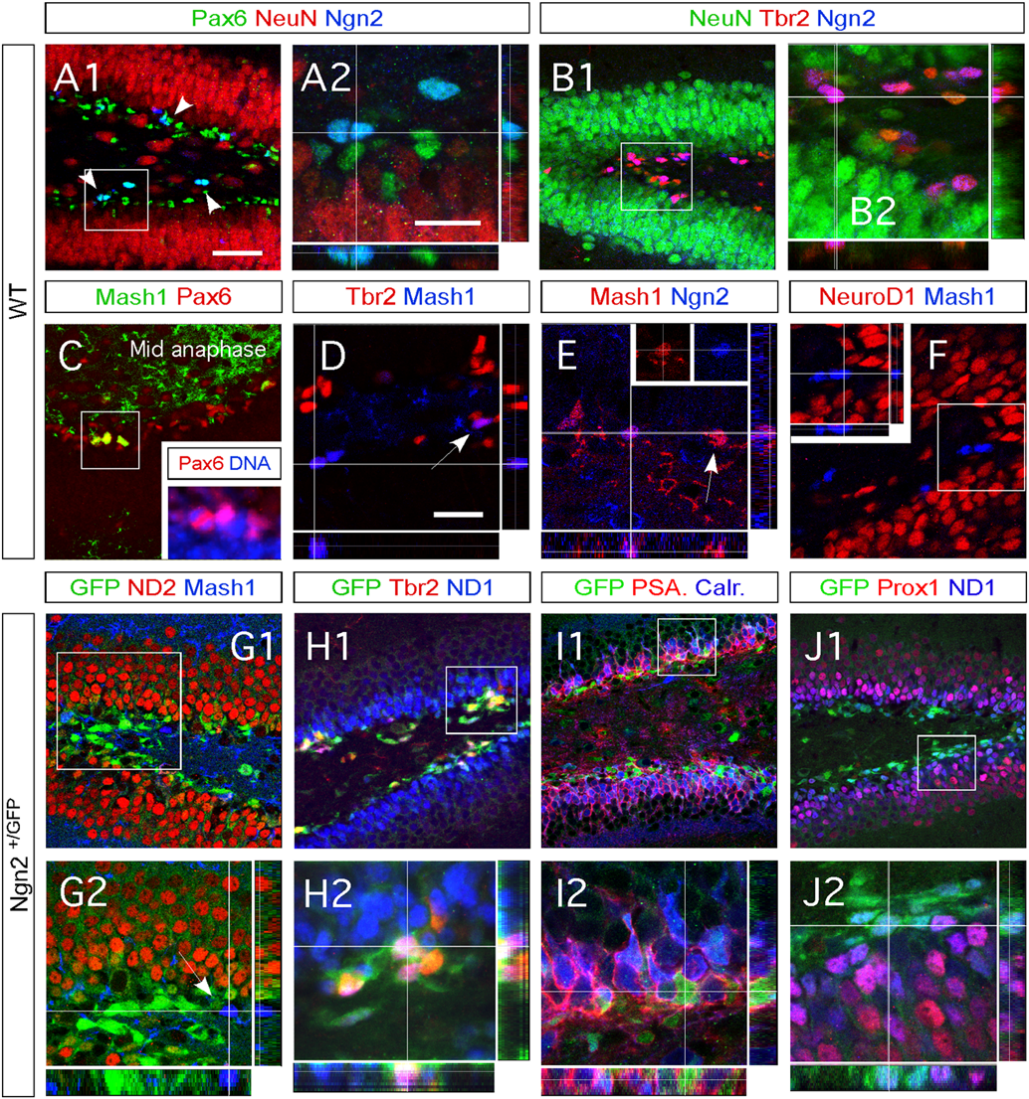
Ngn2+ cells become hippocampal granule neurons. (A1–J2) Indirect immunofluorescence performed on coronal sections from two-weeks old WT and Ngn2^+/GFP^ mice. (A1–A2) Ngn2-expressing cells co-express Pax6 (>90%), but not NeuN. Ngn2+ cells are often observed by doublet of cells. (B1–B2) Almost all Ngn2+ cells express Tbr2, however only half of Tbr2+ cells co-express Ngn2. (C–F) Mash1 expression precedes that of Ngn2. (C) All Mash1+ cells express Pax6, and exhibit high mitotic activity (See also supplementary Figure 1J). Mash1 expression ceased at the onset of Tbr2 (D) or Ngn2 expression (E). (F) Mash1 cells never co-expressed NeuroD1. (G1–G2) GFP (driven by the Ngn2 promoter) starts upon Mash1 downregulation. Mash1 expression and GFP expression do not co-localize. Ngn2 GFP-expressing progenies become DG granule neurons, the latter expresses NeuroD2 during their maturation. Ngn2 GFP-expressing progenies sequentially express Tbr2 and NeuroD1 (H1–H2), the cellular membrane markers PSA-NCAM and Calretinin (I1–I2) and Prox1, an identity marker of mature hippocampal granule neurons (I1–J2). (K) Expression patterns of cellular markers and transcription factors during hippocampal DG granule neurogenesis. The type-2a stage has been divided into early and late type-2a stages, based on the sequential expression of Mash1 and Ngn2. Panels A2, B2, G2–J2 represent a higher magnification of framed areas in corresponding panels A1, B1, G1–J1. The white crosshairs in these panels were positioned to show co-expression of the markers of interest in single cells. Rectangular images on the bottom and right of the panels A2, B2, D–F and J2–G2 represent a projection of 14-Z stacks images (total of 10–20 µm thick). Scale bars: 50 µm (A1, B1, C, G1, H1, I1 and J1), 25 µm (A2, B2, D, E, F, G2, H2, I2 and J2).

We first confirmed that stem cells in the SGZ exhibit characteristics of radial glia [3], [11], [16]. They extend radial processes from the SGZ to the apex of the subgranular layer (SGL) and the basal molecular layer. Moreover, they express the intermediate filament protein nestin, glial fibrillary acidic protein (GFAP) and glial glutamate transporter (GLAST) [16] (Figure 1A and B and figure S1A and S1B). The GFAP- and GLAST+ cells are defined as type-1 cells [3]. They also express the paired-homeodomain transcription factor Pax6 (Figure 1B). These radial glia-like stem cells divide relatively infrequently, and are believed to undergo symmetric division (giving rise to two identical stem cells) under some conditions. They can also divide asymmetrically and give rise to a new stem cell and one neuronal progenitor, which usually is defined as a type-2 cell (Figure 2B) [3].

Type-2 cells are divided into two different populations: type-2a expressing nestin and type-2b co-expressing nestin and doublecortin (Dcx) [23]. Based on a previous report [24], we examined the expression pattern of the T-domain transcription factors Tbr1 and Tbr2. We found that Tbr2 is expressed in type-2a cells, in agreement with a recent report [25]. In the SGZ of 2 week-old mice, we found an average of 50–70 Tbr2^+^ cells per 30 µm-thick section throughout the rostro-caudal axis of the dorsal hippocampus. This number was reduced by 35% in 2 month-old mice (Figure 1C with figure S1C and data not shown). As opposed to Tbr1 (Figure 1D and figure S1D), Tbr2^+^ cells did not co-express Dcx or PSA-NCAM (Figure 1C and figure S1C). We observed that Tbr1-immuoreactivity gradually decreased as neurons matured and started to express NeuN (Figure 1H and figure S1H). Thus, Tbr2 labels type-2 cells, while Tbr1 is expressed by immature granule neurons.

We then examined the molecular phenotype of type-3 cells, a cell type that transiently expresses Calretinin [26]. We hypothesized that hippocampal type-3 cells might also express the bHLH transcription factor NeuroD1, known to partially overlap with Tbr2 and Tbr1 in different brain regions [24]. We found that NeuroD1 expression in the hippocampus starts in Tbr2+ cells and extends to post-mitotic Tbr1+ cells, which also express the hippocampal granule identity transcription factor Prox1 (Figure 1G and 1I and figure S1G and S1I). While Tbr1 expression ceases during granule maturation, that of NeuroD1 is weakly maintained when NeuN expression starts (Figure 1G and 1H and figure S1G and S1H). We found that calretinin expression decreased during granule neuron maturation, before NeuroD1 was reduced (Figure 1F). Thus NeuroD1 is expressed in type-2b and type-3 cells, as well as immature granule neurons. Finally, we found that NeuroD2 starts to be expressed just after NeuroD1 and, unlike NeuroD1, continues to be highly expressed in mature neurons (Figure 1J and data not shown).

### Mash1 and Ngn2 define the early versus late type-2a stage in 2 weeks old mice

Having established the hierarchy of transcription factors and cellular markers appearing during hippocampal neurogenesis, we set out to clarify the position of the two bHLH transcription factors Ngn2 and Mash1, in this process. We found that more than 90% of Ngn2-immunoreactive cells are Pax6 positive (Figure 2A), and almost all co-express Tbr2 (Figure 2B). Thus, Ngn2 appears to be expressed by type-2a cells. As our antibodies against Ngn2 and NeuroD1 were made in the same species, we could not determine if there was an overlap between these two proteins. However, by comparing their overlapping expression pattern with that of Tbr2, we propose that some Ngn2+ cells co-express NeuroD1, and that they appear at the onset of NeuroD1 expression and downregulation of Ngn2. We base this assumption on the facts that more than 50% of the Tbr2+ cells are Ngn2+ and more than 50% of Tbr2+ cells also express NeuroD1. The expression of Ngn2 in adult hippocampus was hard to detect using immunohistochemistry due to the low expression of the protein (data not shown). However, it is present, as previously described using reporter mice and as revealed by in situ hybridization [21].

We next examined the expression of Mash1 and compared it with that of Ngn2, Pax6 and Tbr2. We found some Mash1+ cells (10–20 cells per section) in the dorsal hippocampus. Almost all of them co-expressed Pax6 (Figure 2C). Only few cells co-expressed Tbr2 (Figure 2D) or Ngn2 (Figure 2E), suggesting that Mash1 is downregulated when these two proteins are expressed. None of the Mash1-expressing cells were positive for NeuroD1 (Figure 2F). Mash1+ cells were still undergoing mitosis (Figure 2C) and consequently over 90% of them co-labeled with the cell-cycle marker Ki67 (Figure S1J).

Taken together, we show that Mash1 and Ngn2 are expressed in early and late stages of maturation of type2a progenitors, respectively, and that both transcription factors are co-expressed briefly when cells transit from early to late phase type2a cells.

### Ngn2 progenies become hippocampal granule neurons

Previous work has shown that in Mash1 null mutant mice hippocampal neurogenesis is not reduced and the hippocampus is not malformed [20]. Therefore we focused our initial analysis on the role of Ngn2 in hippocampal neurogenesis. We characterized the fate of Ngn2-expressing cells using Ngn2 Knock-in green fluorescent protein (GFP) mice (Ngn2^+/GFP^) [27]. In the SGZ of 2 week-old hippocampi, we found bright GFP-expressing cells that were positive for Tbr2 (Figure 2H), the immature neuronal marker PSA-NCAM (Figure 2I) and NeuroD1 (Figure 2J). Furthermore, Prox1+, NeuroD2+ and calretinin+ cells were also weakly positive for GFP. We made similar observations in 2 month-old mice (data not shown) confirming earlier published results [21]). We did not observe Mash1^+^/GFP^+^ and NeuN^+^/GFP^+^ cells (Figure 2G). These data from Ngn2^+/GFP^ mice are entirely consistent with our earlier observations using immunocytochemistry to label Ngn2-expressing cells and with GFP undergoing slow degradation after the expression of Ngn2 has ceased. Importantly, none of the GFP-expressing cells, including those only weakly fluorescent, residing in the SGL expressed the astrocytic marker GFAP or the oligodendrocytic markers CNPase (data not shown). Taken together, the data show that Ngn2 progenies become neurons and never generate astrocytes or oligodendrocytes.

### Marked reduction of hippocampal granule neurons in absence of Ngn2

**Figure 3:**
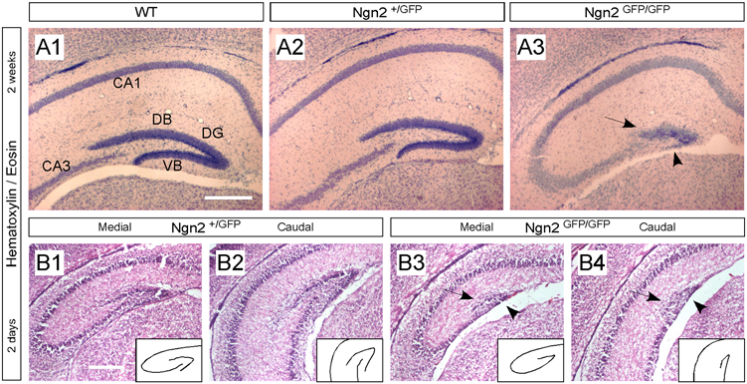
Malformation of the hippocampal DG structure in Ngn2 null mutant animals. (A1–A3) Hematoxylin/Eosin staining performed on coronal sections from two-weeks old WT, Ngn2^+/GFP^ and Ngn2^GFP/GFP^ mutant animals, shows that the loss of Ngn2 results in the formation of a smaller hippocampus and primitive and undeveloped DG. (B1–B4) The malformation of the DG in Ngn2^GFP/GFP^ mutant animals can already be observed two days after birth, throughout the hippocampal structure, along the rostro-caudal axis (see also supplementary figure 2). The inlets of rectangular panels in bottom right corners show a schematic drawing of the shape of the DG for Ngn2^+/GFP^ and Ngn2^GFP/GFP^ mutant animals; black arrowheads point to the undeveloped and missing dorsal blade and ventral blade of the DG, respectively, in Ngn2^GFP/GFP^ mutant animal. Scale bars: 200 µm (A1–B4).

To determine the role of Ngn2 during the initiation of hippocampal granule neurogenesis, we analyzed the hippocampus of mice lacking Ngn2 (Ngn2^GFP/GFP^). In contrast to the mice use by Galichet and coworkers, our mice have a lifespan of only 2 weeks after birth. Therefore, we analyzed 2 week-old and younger mice. Consistent with previous observations [20], the hippocampus in Ngn2^GFP/GFP^ mutants is clearly malformed (Figure 3A). In two week-old Ngn2^GFP/GFP^ mice, the ventral blade of the DG is completely absent. Already on postnatal day 1–2, the ventral blade of the DG is malformed along its whole rostro-caudal axis (Figure 3B).

**Figure 4:**
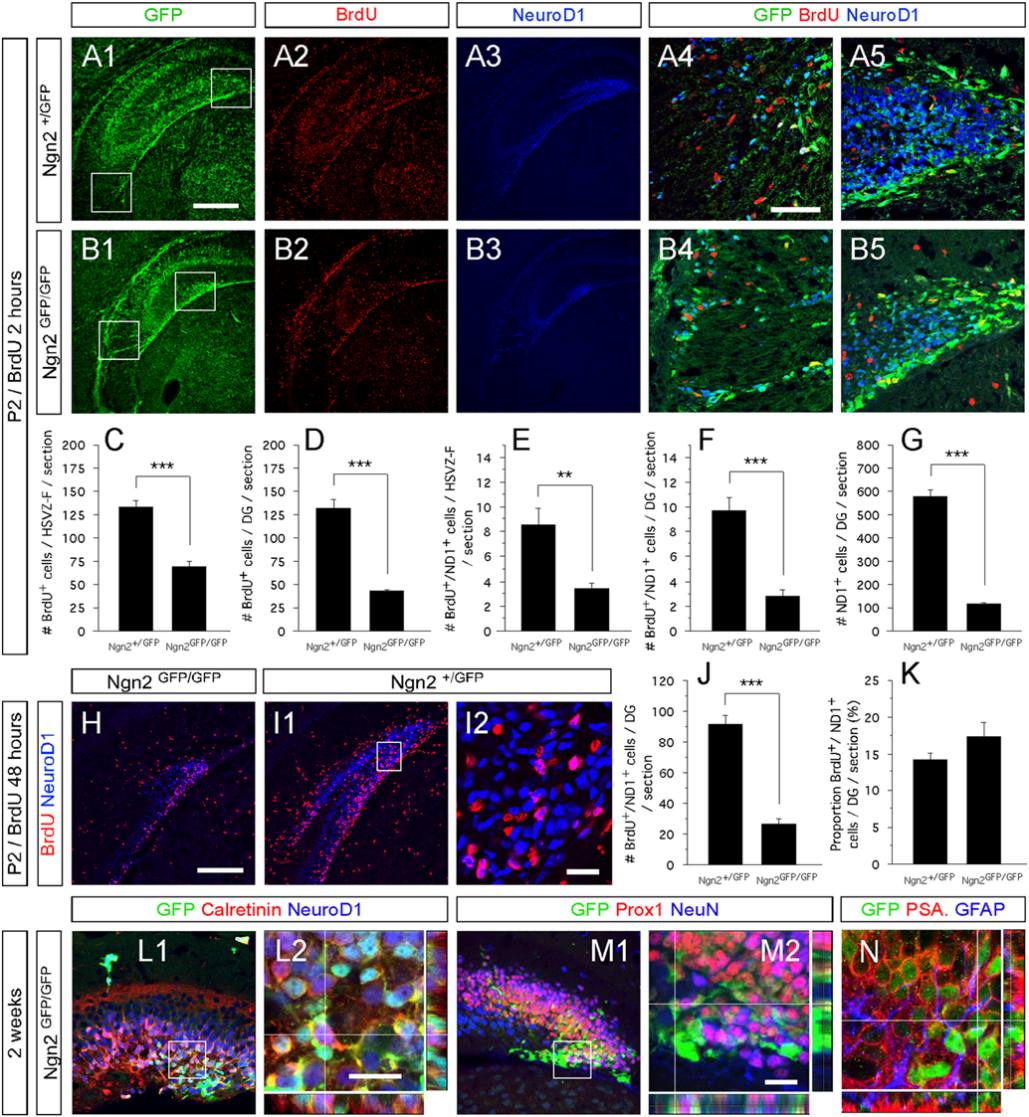
Ngn2 directs the production of hippocampal DG granule neuroblasts. (A1–B5, H–I2 and L1–N1) Indirect immunofluorescence performed on hippocampal coronal sections from two-days old Ngn2^+/GFP^ and Ngn2^GFP/GFP^ mutant animals (A1–B5 and H–I2) and two-weeks old Ngn2^+/GFP^ mutant animals (L1–N1). (A1–B5) Presence of BrdU+ cells (red) in the hSVZ, chains of migrating cells and DG of two-days old Ngn2^+/GFP^ and Ngn2^GFP/GFP^ mutant animals injected with BrdU two hours prior to sacrifice. A4, A5, B4 and B5 represent high magnification of the framed areas in A1 and B1, respectively. (C–I2) Cell proliferation and neurogenesis are severely decreased in Ngn2^GFP/GFP^ mutant animals. The histograms C–G and J and K depict the average number or percentage (±SEM); P<0.0001 = *** and P<0.01 = **. (L1–M2) Granule neuron maturation is confirmed by indirect immunofluorescence on hippocampal sections from two-weeks old Ngn2^GFP/GFP^ null mutant animals, as revealed by the co-expression of Calretinin, NeuroD1, Prox1 and NeuN in GFP-expressing cells. (N) In the absence of the Ngn2 protein, GFP-expressing cells do not switch from a neuronal commitment to an astrocytic phenotype. Rectangular images on the bottom and right of the panels L2, M2 and N represent a projection of 14-Z stacks images (total of 10–14 µm thick). The white crosshairs in these panels were positioned to show co-expression of markers of interest in single cells, as labeled above each panel. Scale bars: 200 µm (A1–3 and B1–3), 100 µm (H and H1), 50 µm (A4, A5, B4, B5, L1 and M1), 25 µm (I2, L2, M2 and N).

As neither migration defects nor cell death cause the reduced number of hippocampal neurons in Ngn2 null mice [20], we asked if Ngn2 is necessary for production of hippocampal granule neuroblasts. We first injected 2 day-old mouse pups with BrdU 2 hours prior to sacrifice, allowing us to evaluate cell proliferation and ongoing neurogenesis. We also injected BrdU into female mice on their final day of pregnancy and examined the brains of their pups 48 hours later (corresponding to P1–P2 old pups). This allowed us to assess the number of neurons derived from the last day of intrauterine development. Regardless of whether the mice were WT or hetero/homozygous mutant pups, we found cells that had incorporated BrdU in the hippocampal subventricular zone (hSVZ), fimbria and the DG (Figure 4A and B; [28]), suggesting that progenitors originating in the hSVZ divide while migrating towards the DG. Most of them were organized in chains typical of migrating cells (Figure 4A and B and figure S2). In Ngn2^GFP/GFP^ mutant mice injected with BrdU 2 hours prior to sacrifice, we observed a decrease in the number of BrdU+ cells compared to heterozygous littermates. Thus, the numbers of newborn cells were reduced in the hSVZ/fimbria (133.3±7.5 for Ngn2^+/GFP^ vs 69.7±5.0 for Ngn2^GFP/GFP^) and DG (132.0±9.1 for Ngn2^+/GFP^ vs 42.6±2.2 for Ngn2^GFP/GFP^)(Figure 4C and D). We also examined the number of newborn cells differentiating into neurons, and identified them by the co-expression of BrdU and NeuroD1. As expected, they were relatively few in numbers because the short delay (2 hours) between BrdU administration and sacrifice in this first experimental paradigm. In Ngn2^GFP/GFP^ null mutants, we found the number of cells differentiating into neurons to be reduced by 60% and 71% in the hSVZ/fimbria (8.6±1.4 for Ngn2^+/GFP^ vs 3.4±0.5 for Ngn2^GFP/GFP^) and DG (9.7±1.1 for Ngn2^+/GFP^ vs 2.8±0.6 for Ngn2^GFP/GFP^), respectively, (Figure 4E and F). As a result, the number of NeuroD1+ cells in the DG was reduced by 80% (580.3±27.7 for Ngn2^+/GFP^ vs 116.2±7.3 for Ngn2^GFP/GFP^; Figure 4G).

We obtained similar results in the second paradigm, i.e. in mice that we sacrificed 48 hours after BrdU administration and in which a larger number of the newborn cells had time to mature into neurons. In this case, the number of BrdU^+^/NeuroD1^+^ cells in the DG was reduced by 73% of Ngn2^GFP/GFP^ mutant animals (91.5±24.1 for Ngn2^+/GFP^ vs 26.4±3.3 for Ngn2^GFP/GFP^) (Figure 4J). We found no differences in the proportion of BrdU+ cells that expressed NeuroD1 in Ngn2^+/GFP^ and Ngn2^GFP/GFP^ mice (14.2±1.0% and 17.4±1.8% respectively; Figure 4K). This shows that the few cells that manage to proliferate in the Ngn2 null mice have the same ability to differentiate into neurons as those in mice with one Ngn2 allele.

Interestingly, the absence of Ngn2 did not alter the identity of neurons in the DG granule layer in 2 week-old Ngn2 null mice. The maturing granule progenitors sequentially expressed Pax6, Tbr2 (Figure S3A and S3B), NeuroD1 and Calretinin (Figure 4L), PSA-NCAM (Figure 4N), Tbr1 (data not shown) and Prox1 (Figure 4M). We confirmed that the same transcriptional cascade is active in the 20% (compared to mice with one Ngn2 allele) residual granule neurons that are formed two day-old in Ngn2^GFP/GFP^ mice (Figure S3D–G and data not shown).

We next investigated if the cells that failed to develop into neurons in Ngn2^GFP/GFP^ mutant mice, became glial cells. We found that cells expressing GFP never co-labeled with the astrocyte marker GFAP (Figure 4N) or the oligodendrocyte marker CNPase (data not shown).

Altogether, our results show that Ngn2 plays an important role during the production/amplification of hippocampal granule neuroblasts, but not the acquisition of the granule neuron identity (Figure S7).

### Ngn2 controls the amplification of granule neuron progenitors

**Figure 5:**
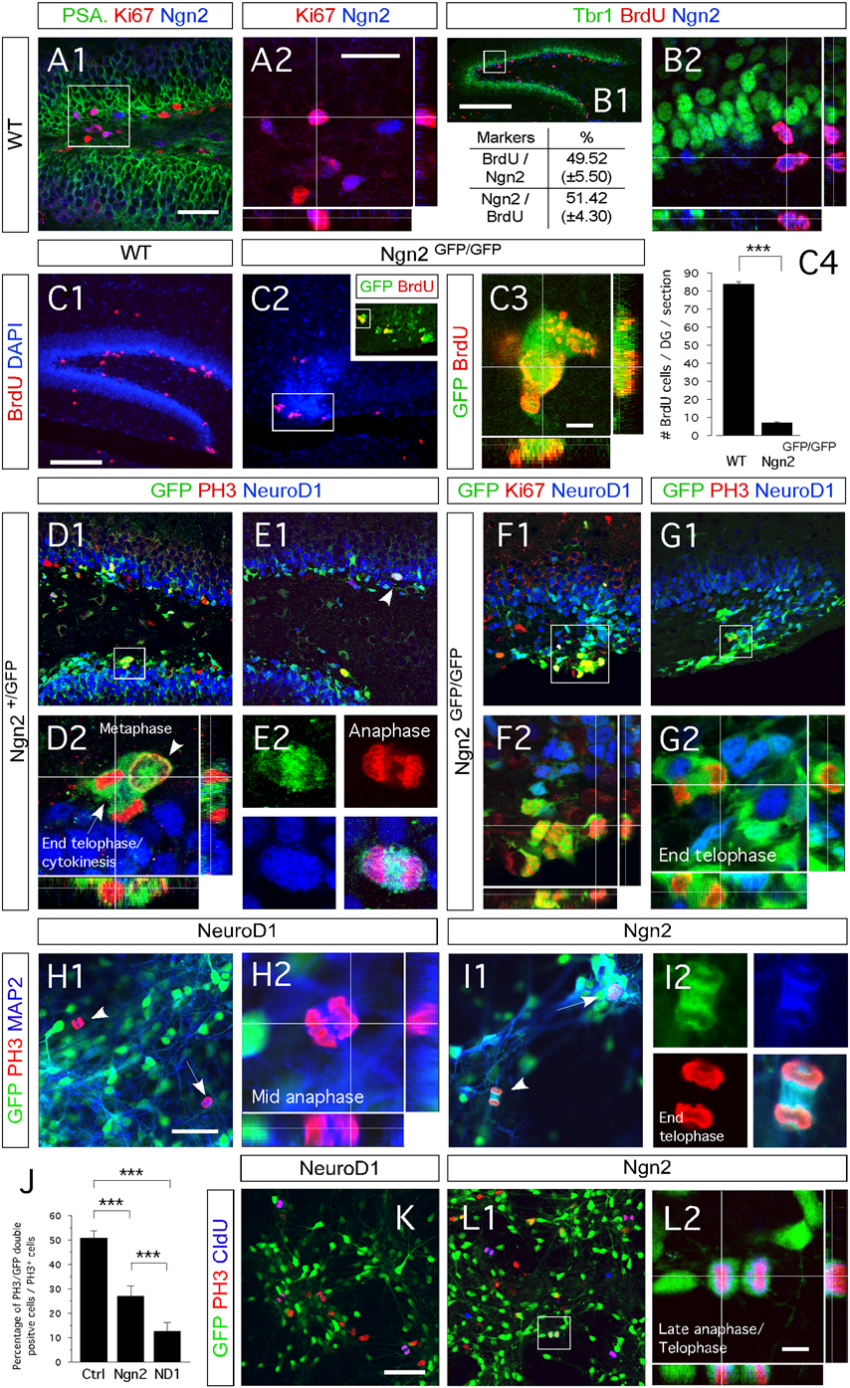
Ngn2 and NeuroD1 mark the initiation and end of amplification of hippocampal DG granule neuroblasts, respectively. (A1–C3 and D1–K2) Indirect immunofluorescence performed on hippocampal coronal sections from postnatal two-weeks old WT, Ngn2^+/GFP^ and Ngn2^GFP/GFP^ mutant animals and Ngn2 and NeuroD1 retrovirally transduced E14.5 cortico-hippocampal neurospheres. (A1 and A2) Fifty percent of Ngn2+ cells co-label with Ki67. (B1 and B2) One BrdU injection with a two-hour chase labels 50% of the Ngn2-expressing cell population, representing half of the total number of proliferating cells in the DG. (C1–C4) In the DG of two-weeks old Ngn2^GFP/GFP^ mutant animals, the number of dividing cells is substantially decreased compared to WT (data expressed as ±SEM; *** P<0.0001). (D1 and D2) In Ngn2^+/GFP^ mice, GFP/Ngn2-expressing cells undergo division and give rise to GFP^+^/NeuroD1^−^ cells. These cells have a baso-apical orientation. (E1 and E2) GFP/Ngn2-expressing cells stop dividing when NeuroD1 expression starts. Dividing cells expressing GFP and NeuroD1 have a planar orientation (see also supplementary figure 4). (F1 and G2) All cells brightly GFP present in the ventral blade of the DG of Ngn2^GFP/GFP^ mutant animals co-express Ki67; however, only very few divide, indicating that in absence of Ngn2, Ngn2 progenies stay arrested in the cell cycle. (H1–J) Presence of GFP+/MAP2+/PH3+ dividing neuroblasts in Ngn2-transduced E14.5 hippocampo-cortical neurospheres compared to NeuroD1-transduced cultures. (K–L2) Five days differentiated Ngn2 transduced cells (GFP+) that have been cultures for two more additional days in medium supplemented with CldU, are still capable of dividing after 7 days, as opposed to NeuroD1 transduced cells. Rectangular images on the bottom and right of the panels A2, B2, C3, D2, F2, G2, I1 and J1 represent a projection of 14-Z stacks images (total of 10–14 µm thick) from framed areas or pointing arrows in panels A1, B2, C3, D2, F2, G2, I1 and J1, respectively. The white crosshairs in these panels were positioned to show co-expression in single cells of markers of interest, as labeled above each panel. Arrows and arrowheads point at cells of interest. Scale bars: 100 µm (B1, C1 and C2), 50 µm (A1, D1, E1, F1, G1, H1, I1, K and L1), 25 µm (A2, B2, and F2), 10 µm (L2), 5 µm (C3, E2, G2, H2 and I2).

We next monitored the mitogenic activity of Ngn2+ cells in the DG of 2 week-old WT mice. We observed that over 50% of Ngn2+ cells in the DG of WT mice co-expressed the mitosis marker Ki67 (Figure 5A). Inspired by this finding, we injected BrdU into 2 week-old WT and Ngn2^GFP/GFP^ mice and compared cell proliferation in the DG. We observed 92% reduction in number of proliferating cells in the SGZ of Ngn2 null mutant mice (6.7±0.5) compared to WT (83.6±1.2) and heterozygotic (data not shown) littermates (Figure 5B–C). This data confirm the importance of Ngn2 during the amplification of granule progenitor.s.

To further explore whether Ngn2+ cells become post-mitotic or still proliferate after Ngn2 is downregulated, we performed immunohistochemistry on sections through the DG of Ngn2^+/GFP^ mice. We stained them with the mitosis marker Phospho-histone 3 (PH3) and NeuroD1, which is downstream of Ngn2 in the transcriptional cascade controlling neurogenesis. Thus, in the same sections we could identify whether cells that had initiated Ngn2 expression (GFP labeled), continued to divide (PH3+) or committed to neuronal differentiation (NeuroD1+). We observed some GFP+ cells that co-expressed PH3. They all exhibited morphological characteristics of one of the five mitotic phases: prophase, metaphase, anaphase, telophase and cytokinesis (Figure 5D and data not shown). Unexpectedly, cells that colabeled for GFP, PH3 and NeuroD1 (Figure 5E and figure S4A and S4B) were rare. This indicates that Ngn2+ cells undergo division/amplification and that they mature into post-mitotic neurons upon NeuroD1 expression. In mice lacking Ngn2, we found the cells expressing GFP localized to the malformed ventral blade of the DG (Figure 5F and G). All of these cells were Ki67+ and they only very rarely expressed PH3 (Figure 5F and G, and figure S4C) in the absence of Ngn2 most of the cells are arrested in the cell cycle prior to entering the M phase and their mitosis is impaired. These data suggest that the mechanism of action of Ngn2 is conserved from development of the DG to postnatal hippocampal neurogenesis.

Our findings suggest that Ngn2 regulates amplification and cell cycle exit of DG granule progenitors. To examine this hypothesis, we compared the effects of Ngn2 and NeuroD1 on mitotic activity in embryonic cortico-hippocampal neurosphere-derived progenitors, 5 days upon transduction, in vitro. In cultures transduced with Ngn2 retrovirus, we found that 27.2% (±4.1%) of the PH3+ cells had been transduced (Figure I1–J). These cells were immunopositive for MAP2 (Figure 5I1 and I2) and therefore represented dividing neuroblasts. By contrast, in NeuroD1 transduced cultures, only 7.3% of PH3+ cells were GFP+ (Figure 5H1, H2 and J). All of the NeuroD1-transduced cells became MAP2+ (Figure 5H). To confirm this data we pulse-labeled transduced cultures with chlorodeoxyuridine (CldU) for 48 hours, five days after differentiation. In contrast to NeuroD1-transduced cultures, we observed dividing cells transduced with Ngn2 retrovirus that were positive for PH3 and that had incorporated CldU (Figure 5K–L2). As the number of cells PH3+/CldU+/GFP+ we observed was low, we did not quantify this finding.

Collectively, we have shown that Ngn2 is required for granule neuroblast production and amplification and that in the absence of Ngn2 the progenitors arrest in the cell cycle. NeuroD1, on the other hand, induces cell cycle exit and promotes rapid neuronal maturation.

### NeuroD1 directs neuronal differentiation and maturation

**Figure 6:**
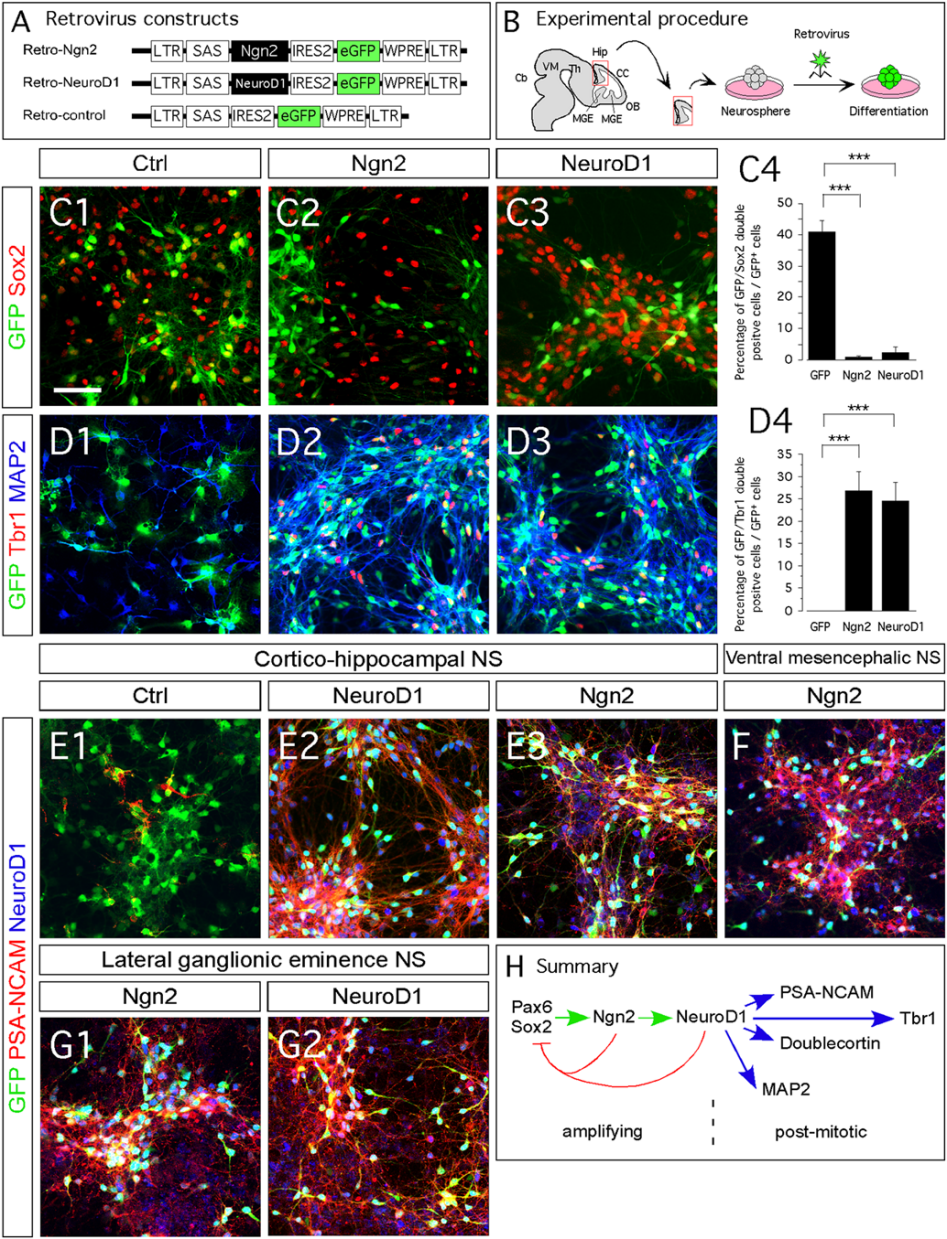
Ngn2 induces NeuroD1 expression, in vitro. (C1–G2) Indirect immunofluorescence performed on cortico-hippocampal, ventral mesencephalic and lateral ganglionic eminence neurospheres derived from tissue of E14.5 rat embryos. (A) Schematic representation of the retroviral constructs used in this study. (B) Schematic representation of the experimental design. (C1–C4) Upon five days of differentiation, Ngn2- and NeuroD1-transduced cells (GFP+) downregulate the immature neural marker Sox2 (red), as compared to control transduced cells. (D1–D4) Ngn2 and NeuroD1 overexpression induce Tbr1 expression and MAP2. Data in C4 and D4 are expressed as ±SEM; *** P<0.0001 (E1–F) Overexpression of Ngn2 in cortico-hippocampal and ventral mesencephalic neurospheres induces the expression of NeuroD1 and leads to neuronal differentiation, as marked by the expression of PSA-NCAM (red). (G1–G2) The ectopic overexpression of Ngn2 induces ectopic expression of NeuroD1 and neuronal differentiation in LGE neurospheres. (H) Schematic illustration of molecular events upon Ngn2 induction, in vitro. Scale bars: 50 µm (C1–3, D1–3, E1–G2).

Based on our previous observations we proposed that Ngn2 primarily controls amplification of granule neuroblasts and NeuroD1 directs neuronal differentiation. To test this hypothesis, we overexpressed Ngn2 or NeuroD1 in E14.5 cortico-hippocampal neurospheres and compared their effects after 5 days of differentiation of the progenitors. All cortico-hippocampal progenitors expressed Pax6 prior to differentiation (Figure S5). After 5 days, both factors suppressed Pax6 and Sox2 (Figure S5, figure 6C) and induced expression of Tbr1, Map2, NeuroD1 and PSA-NCAM (Figure 6D and E).

Interestingly, all Ngn2-overexpressing cells co-expressed NeuroD1. Based on these findings, we next explored whether Ngn2 overexpression induces NeuroD1 expression, and if NeuroD1 in turn directs neuronal differentiation. We compared the effects of both transcription factors in progenitors that either do or do not normally express them. Thus, we expressed Ngn2 in E14.5 neural progenitors isolated from three different brain regions: cortex/hippocampus, lateral ganglionic eminence (LGE) and ventral mesencephalon (VM). Ngn2 expression in the developing forebrain is normally limited to the neocortex, and it does not appear in LGE tissue [29]. In the developing VM, both Ngn2 and NeuroD1 are expressed, but not when neural progenitors from this region are cultured *in vitro* [30], [31]. In our experiments, cortico-hippocampal progenitors could differentiate into neurons, although they did not normally express NeuroD1 (Figure 6E). When we overexpressed Ngn2 in VM and LGE progenitors, the cells started to express both NeuroD1 and PSA-NCAM (Figure 6F and G). We then overexpressed NeuroD1 in the same types of cultured progenitors and found that the cells became immunoreactive for PSA-NCAM (Figure 6G and data not shown). Thus, NeuroD1 is sufficient to direct neuronal differentiation in cortico-hippocampal-, LGE- and VM-derived progenitors.

Our results from *in vitro* cultures and the analysis of the DG of Ngn2 mutant animals collectively show the neuron-inducing effect of NeuroD1 in hippocampal granule cell progenitors [32].

### NeuroD1 directs exclusive neuronal differentiation of hippocampal granule neuron progenitors

**Figure 7:**
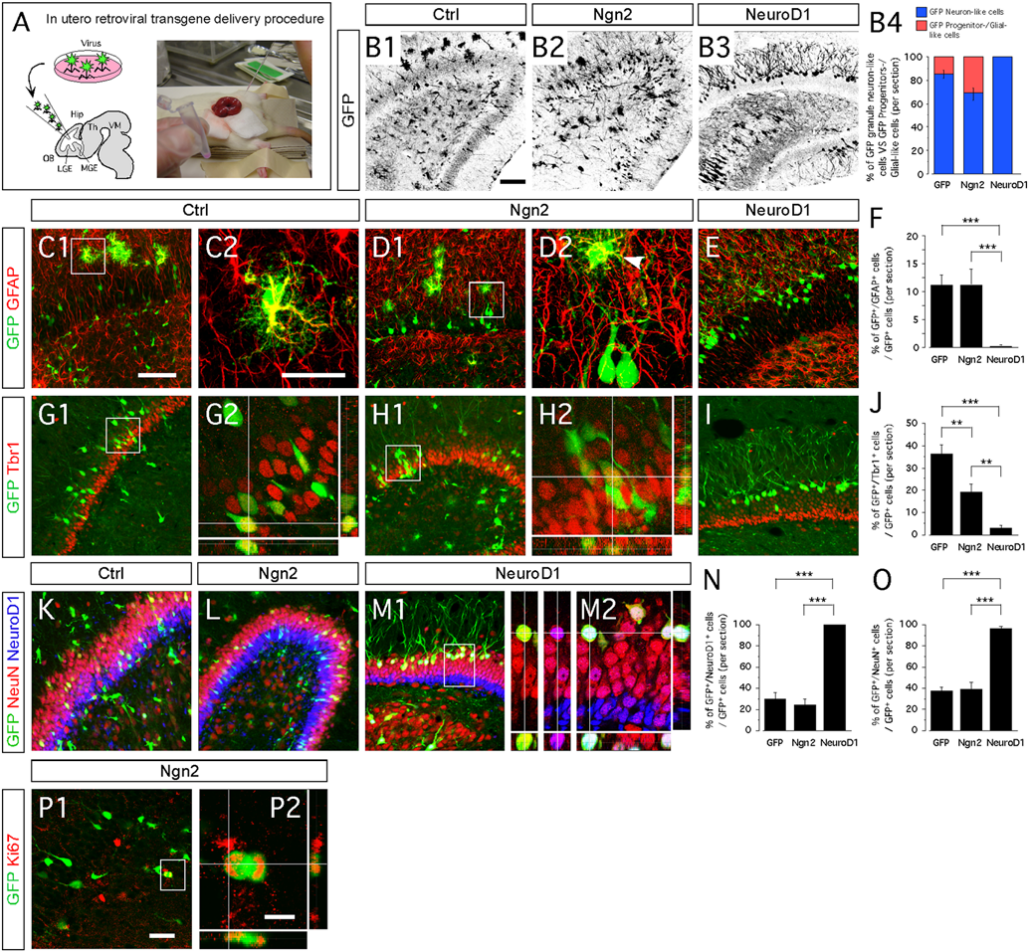
NeuroD1 directs neuronal differentiation of hippocampal progenitors in vivo. (B1–P2) Indirect immunofluorescence performed on hippocampal coronal sections of two-weeks old rats that received a retroviral injection with Ngn2, NeuroD1 or control retroviruses, at the intra-uterine age of E15.5. (A) Schematic representation of the experimental design for in utero retroviral transgene delivery procedure. (B1–B3) Presence of GFP-expressing cells in the hippocampus, three weeks after retroviral injection with Ngn2, NeuroD1 or control vectors. (B4) The proportion of GFP-granule neuron-like cells (blue) and GFP-progenitors/glial-like cells (red) in animals injected with each retrovirus (expressed in percentage of GFP cells) indicate a complete differentiation of NeuroD1-transduced hippocampal progenitors into granule neurons-like cells. (C1–F) Ngn2 overexpression in vivo results in the generation of GFAP-expressing astrocytes, also observed in control group. NeuroD1 overexpression did not induce astrocytic differentiation. (G1–J) In utero injections of control and Ngn2 retroviruses result in the generation of Tbr1+ cells. Only a few Tbr1+/eGFP+ cells were observed in the hippocampal DG of the animals injected with NeuroD1 retrovirus. (K–O) NeuroD1 overexpression induces exclusive neuronal differentiation, in contrast to Ngn2 overexpression. (P1 and P2) A few GFP+ cells that were transduced with Ngn2 retrovirus are found still dividing. Data are expressed as ±SEM; *** P<0.0001 and ** P<0.01. Scale bars: 50 µm (B1–3, C1, D1, E, G1, H1, I, K, L, M1 and P1), 25 µm (C2, D2, G2, H2 and M2), 10 µm (P2).

As the next step, we tested the effects of NeuroD1 *in vivo* and compared them with those of Ngn2. We injected retroviruses carrying the gene for either Ngn2 [33] or NeuroD1, and the reporter gene eGFP into the ventricles E15.5 rat embryos, *in utero* (Figure 7A). Three weeks later, when the rats were about two weeks old, we examined their hippocampi and analyzed the eGFP+ cells. The rats injected with control vector exhibited eGFP+ cells within the hippocampus that were either star-shaped, progenitor/glial-like cells (14.8±3.4%) or neuron-like cells with long neurites (85.3±3.4%) (Figure 7B). In rats that we had injected with the vector encoding Ngn2, the eGFP-labeled, the transduced hippocampal cells were composed of 31.9% (±5.7%) progenitor/glia-like cells and 69.4% (±6.1%) neuron-like cells (Figure 7B). The glia-like subpopulation was immunopositive for the astrocytic marker GFAP (Figure 7C–D). In contrast, virtually all of the transduced cells in rats injected with the NeuroD1 vector became neuron-like cells (99.9%±0.1; Figure 7B). None of these cells stained for GFAP (Figure 7E and F). They were positioned in the external layers of both the ventral and dorsal blades of the DG (Figure 7B3, E and I and figure S6A).

When we examined neuronal maturation of Ngn2- and NeuroD1-transduced cells, we observed that only a few of those transduced with the NeuroD1 vector expressed the early mature neuronal marker Tbr1 (3.1±1.4%; Figure 7I and J). They were located within the basal layers, near the SGL, of the ventral and dorsal blades of the DG. In contrast, a greater proportion of the cells transduced with Ngn2 still expressed Tbr1 (19.6%±3.3%; Figure 7H and J), indicating that they were less mature than the vast majority of the NeuroD1-transduced cells. Immunohistochemistry for NeuN confirmed these data (Figure 7M and O, and figure S6A). Indeed, when we overexpressed Ngn2 the proportion of hippocampal progenitors that became NeuN+ was no greater than in rats transduced with the control virus (Figure 7F).

As a whole, our *in utero* injection experiments confirm that NeuroD1 has a stronger neuron-inducing effect than Ngn2 when overexpressed in hippocampal progenitors.

### Involvement of Mash1, Ngn2, Tbr and NeuroD proteins during human hippocampal neurogenesis

**Figure 8:**
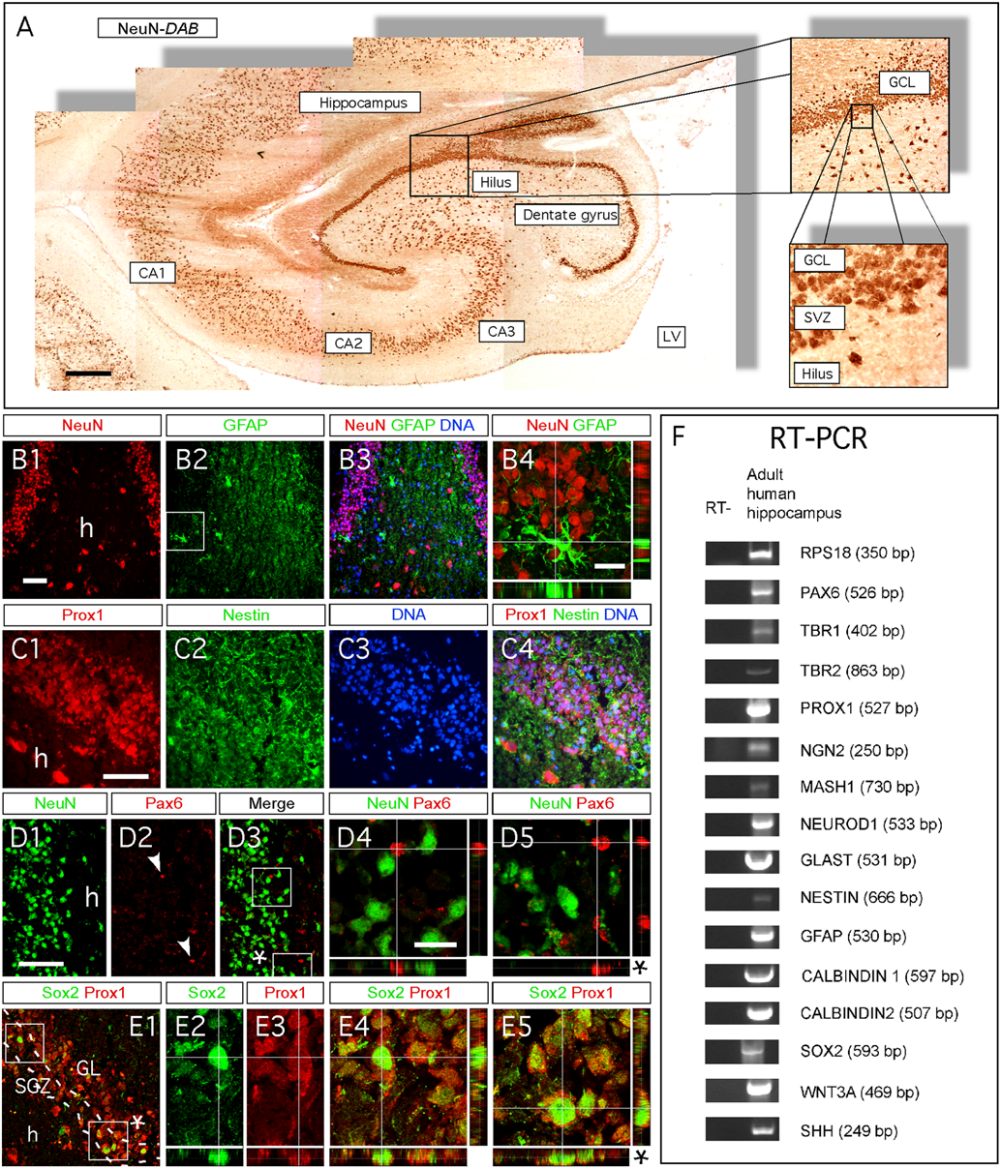
The expression pattern of markers for cell maturation during rodent hippocampal neurogenesis is conserved during human hippocampal neurogenesis. (A–E5) Indirect immunohistochemistries performed on human hippocampal coronal sections of 58-years-old individual. (A) DAB immunohistochemistry for the neuronal marker NeuN shows the neuro-anatomical structure of the hippocampus. (B1–B4) Mature granule neurons and astrocytes/radial glia-like cells express NeuN (green) and GFAP (red), respectively. (C1–C4) Neurons located in the DG express the granule neuron identity marker Prox1. (D1–D5) Pax6 and NeuN expression do not co-localize, suggesting the existence of astrocytes/radial glial stem cell in human hippocampus. (E1–E5) The presence of Sox2+ cells in the SVZ of the DG suggests the occurrence of ongoing neurogenesis. (F) The existence of radial glia stem cells in the human hippocampus is identified by RT-PCR for the markers Pax6, GFAP and GLAST. The molecular bibliography of cellular markers and transcription factors expressed during hippocampal neurogenesis in rodents is conserved during hippocampal neurogenesis in human. Rectangular images on the bottom and right of the panels B4, D4 and D5, E4 and E5 represent a projection of 14-Z stacks images (total of 10–14 µm thick) from framed areas or pointing arrows in panels B2, D3 and E1. The white crosshairs in these panels were positioned to show co-expression in single cells of markers of interest, as labeled above each panel. Arrows and arrowheads point at cells of interest. CA = cornu ammonis, LV = lateral ventricle, SGZ = sub granular zone, GCL = granule cell layer, SVZ = subventricular zone, h = hilus. Scale bars: 300 µm (A), 50 µm (B1–3, C1–4, D1–3 and E1), 25 µm (B4, D4, D5 and E2–5).

Finally, we examined whether the transcription factors and cellular markers that are expressed in rodents are also expressed in human hippocampus. We performed immunohistochemistry on aged human hippocampal DG and found GFAP-, Sox2-, Pax6-, Nestin-, Prox1- and NeuN-immunopositive cells. This indicates that radial glia-like stem cells, neural progenitors and mature granule neurons are present in the aged human hippocampus (Figure 8A–E). While Prox1 is found mainly in granule neurons, Sox2 and Pax6 are exclusively localized to cells in the SGZ (Figure 8D and E).

As for aged rodents, we did not observe the presence of Mash1, Ngn2, Tbr2, Tbr1 and NeuroD1 proteins in aged human hippocampus, using immunohistochemistry. However, we could identify the presence of the transcripts, using RT-PCR (Figure 8F). In addition, we detected mRNA for Prox1, Calbindin1 and Calbindin2/Calretinin (Figure 8F). Interestingly, we also observed Sonic Hedgehog and Wnt-3A transcripts, indicating that they are present in the human DG and supporting previous claims that they are important for the regulation of adult hippocampal neurogenesis (Figure 8F; [34], [35]). Finally, we also found mRNA for GLAST, Pax6 and GFAP which suggests that radial glia-like stem cells are present in the adult human hippocampus, and may play a role in adult human hippocampal neurogenesis (Figure 8F; [8]).

Overall, our data show that different proteins known to play key roles in rodent hippocampal granule neurogenesis are present in the adult human hippocampus.

## Discussion

In this study we determine the functions of Ngn2 and NeuroD1 during hippocampal neurogenesis. First, we map the hierarchy of molecular markers of neurogenesis in the DG. Second, we describe that Ngn2 is necessary for granule progenitor production/amplification. Third, we demonstrate that NeuroD1 directs neuronal differentiation of granule progenitors. Finally, we show that different cellular markers expressed during hippocampal neurogenesis in rodents are present in human.

### Sequential expression of different transcription factors and cellular markers during hippocampal neurogenesis

We clarified the pattern of expression of various markers expressed during postnatal and adult hippocampal granule neurogenesis in detail. We found that the transcription factor Pax6 is initially expressed by both radial glia stem cells (type-1) and early amplifying progenitors (type-2a). The next transcription factor to appear in chronology is Mash1. Mash1 expression characterizes the early stage of hippocampal progenitor amplification (early type-2a). The role of Mash1 during hippocampal granule neurogenesis is still unclear. Mash1 null mutant mice do not display any clear malformation of the DG [20]. However, overexpression of Mash1 alone in the DG leads to the generation of oligodendrocytes [36]. Based on these observations, we hypothesize that Mash1-expressing cells are not yet committed towards a granule cell fate. They may still have the potential to generate both neurons and oligodendrocytes, as is the case in the SVZ [37].

**Figure 9:**
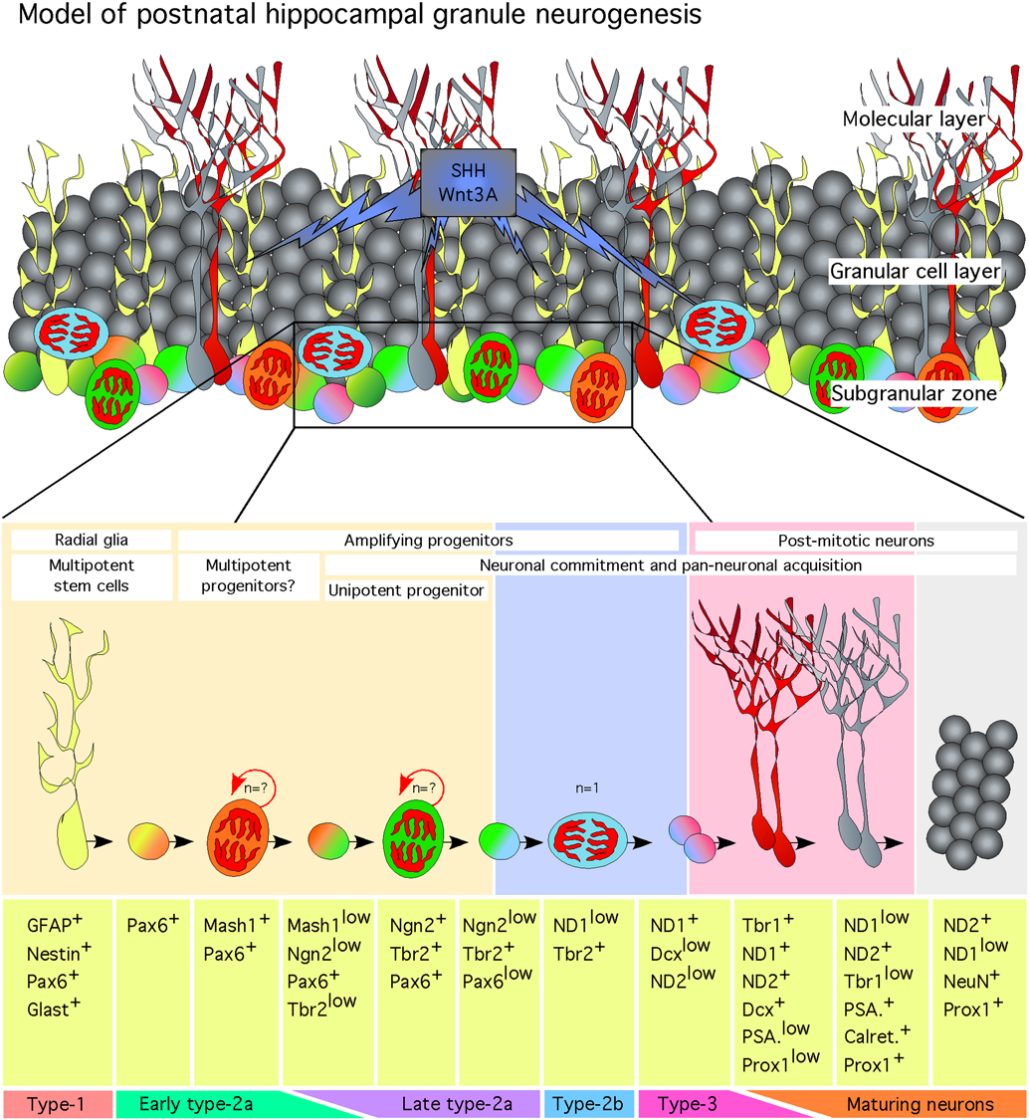
Model of postnatal hippocampal granule neurogenesis. Multipotent GFAP/Nestin/Pax6/Glast+ radial glia stem cells give rise to multipotent and highly dividing Pax6/Mash1+ progenitors. Ngn2 initiates neuronal commitment of Pax6/Mash1+ progenitors. Ngn2 progenies undergo asymmetric divisions and amplify until they divide symmetrically and express NeuroD1. NeuroD1 stops the amplification phase of Ngn2 progenies and direct neuronal maturation. NeuroD1 progenies undergo maturation through the expression of the transcription factors Tbr1, NeuroD2 and Prox1 and the cellular markers Dcx, PSA-NCAM, Calretinin and NeuN.

In agreement with different developing brain regions, we found that Ngn2 starts to be expressed in Mash1-positive transiently amplifying progenitors, i.e. “late type-2a cells” according to the classification we propose. We observed that Pax6 expression persisted longer than that of Mash1, in Ngn2-expressing cells (late type-2a cells), in juvenile but not adult DG (Fig. 1 and figure S1). Later on, Ngn2 is downregulated, whilst Tbr2 expression persists (type-2b cells). The transition from amplifying progenitor to neuroblast is defined by the expression of NeuroD1. Thus, Ngn2 is expressed at the beginning of the transiently amplifying progenitor phase while NeuroD1 marks the end of that period. This applies both to the juvenile and adult rat brain [21], [25]. After NeuroD1 is turned on the progenitors leave the cell cycle, gradually mature, express PSA-NCAM, NeuroD2, Calretinin, Prox1, Tbr1 and, finally, NeuN. NeuroD1 expression persists at low levels in mature neurons (Figure 9; [10]). We found the sequential expression of these markers conserved in juvenile (P2 and 2 weeks) and adult rodent brains. Importantly, we observed that the same transcription factors and neuronal markers are also present in the adult human hippocampus and arranged spatially in a manner reminiscent with what we saw in rodents. It is possible, however, that some of these markers are expressed for longer or shorter periods at postnatal and adult stages of hippocampal progenitor maturation. Moreover, one can ask to which extent the number of divisions occurring during neuroblasts maturation is conserved at postnatal and adult stages of neurogenesis.

### A new function for Ngn2 in maintenance of a progenitor state for granule neuron production/amplification during embryonic and postnatal hippocampal neurogenesis

In agreement with earlier work, we confirm that Ngn2 is indispensable for hippocampal development and plays a vital role in postnatal hippocampal granule neurogenesis. Ngn2 null mutant animals display a reduced size of the cornu ammonis and a malformed DG (Figure 3 and 4; [20]). We show that the number of newborn neurons (incorporated BrdU) is decreased in absence of Ngn2, but hippocampal granule neuron subtype specification is not affected, post-nataly. Likewise, neurons from other brain regions, e.g. ventral midbrain dopamine neurons, can be generated in the absence of Ngn2 [30], [31]. The reduced numbers of mature neurons in the hippocampus and ventral midbrain of Ngn2 null mutant animals does not appear to be due to cell death or migration defects [20], [31]. Instead it may be due to a defect in the generation and/or amplification of neuronal progenitors. Indeed, in the hippocampus of postnatal animals, we observed that cells lacking Ngn2 arrest in the cell cycle, maintain Pax6 expression and cease to proliferate.

When overexpressed in neural progenitors *in vitro*, Ngn2 lead to up-regulation of NeuroD1 and caused neuronal differentiation (Figure 6), which was not always the case when we overexpressed the same factor *in vivo* (Figure 7L, N and O). We also saw that Ngn2 does not always efficiently induce cell cycle exit. Typically some mitotically active eGFP+ cells were present in the DG of rats injected with the Ngn2 retrovirus (Figure 7P), and we observed that cultured Ngn2-transduced cells are still capable of dividing. Thus, Ngn2 does not always promote cell cycle exit and neuronal commitment, but depending on the state of the cells, Ngn2 may instead promote alternate cellular fates [38]–[40]. Indeed, an earlier study has shown that Ngn2-overexpressing progenitors generate oligodendrocytes when grafted to the adult spinal cord [40]. We have also seen that Ngn2-overexpressing neural progenitors from the embryonic midbrain form astrocytes when grafted to the striatum (unpublished observations). Probably, Ngn2 cannot influence already committed cells, but rather would control neuroblasts production. One could speculate that Ngn2 oscillates during granule neuron formation, as recently demonstrated during neocorticogenesis [41], [42].

### NeuroD1, but not Ngn2, is obligatory for granule neuron progenitor differentiation

Hippocampal granule progenitors can mature and express Tbr1 and Prox1, both during the development and postnatally, even in the absence of Ngn2. Because neuronal differentiation still occurs in Ngn2 null mice, it is clear that compensatory, Ngn2-independent mechanisms induce NeuroD1 expression. One candidate is Ngn1, which is expressed during neocorticogenesis, the specification of olfactory sensory neurons and during embryonic rat hippocampal development [43]–[47]. Both Ngn1 and Ngn2 regulate NeuroD1 [19], [48]. The introduction of two mutant forms of Ngn1, a deletion of the basic region of Ngn1 and a substitution of two amino acids in the C-terminal basic region, prevents NeuroD1 expression and neuronal differentiation [48]. The double null Ngn1/Ngn2 mutant displays a more severe phenotype than single gene (Ngn1 or Ngn2) mutant mice. For example, the total brain size of the double mutants is much smaller [10], [32]. If the role of Ngn1 during hippocampal neurogenesis is to activate NeuroD1 and that of Ngn2 is to control granule neuroblasts production/amplification, the DG of double Ngn1/Ngn2 knockout mice should resemble that of NeuroD1 null mutant mice.

We demonstrated that NeuroD1 directs neuronal differentiation both *in vitro* and *in vivo*. In progenitors isolated from different embryonic brain regions and cultured *in vitro*, we found that overexpression of NeuroD1 induced neuronal differentiation. The neurons generated expressed PSA-NCAM, Dcx and MAP2. After *in utero* retroviral vector-mediated gene delivery, virtually all cells transduced with NeuroD1 became neurons. In contrast, progenitors transduced with Ngn2 or control retroviruses adopted a neuron-like morphology in only 70–85% of cases. Under *in vitro* cell culture conditions, when we overexpressed Ngn2 in progenitors derived from the LGE we observed robust expression of NeuroD1 and neuronal differentiation. These *in vitro* results differ from those we obtained when we transduced the embryonic brain with a viral vector expressing Ngn2. In this latter case, Ngn2 did not induce neurons and the transduced cells did not express NeuroD1. Therefore, Ngn2 appears to direct non-neuronal cell type specification *in vivo* [38]–[40]. Taken together, we have confirmed that NeuroD1 plays a key role in neuronal differentiation in the hippocampus, both during development and in the adult brain.

### Concluding remark

We present a detailed classification of different stages in hippocampal neurogenesis. Our detailed molecular mapping of hippocampal neurogenesis allows for a more accurate analysis of how new factors stimulate neurogenesis at different steps in the development of granule neurons. Thereby we hope to facilitate the development of new agents, which stimulate endogenous progenitors in the treatment of diseases.

## Materials and Methods

### Animal tissue preparation

The creation of the Ngn2 transgenic mice was reported elsewhere (ref guillemot). Heterozygote male and female mice were crossed to obtain WT, heterozygote (Ngn2^+/GFP^) and null mutant (Ngn2^GFP/GFP^) animals. Tails of the Ngn2 offspring were used to obtain DNA for determination of the genotype using a polymerase chain reaction (PCR) assay as previously reported [31]. As null mutant Ngn2^GFP/GFP^ do not survive longer than 2.5–3 weeks after birth, Ngn2^GFP/GFP^ and their littermates were sacrificed at the postnatal ages of two days (P2) or two weeks for this study. Neurogenesis was assessed in two weeks or two months old WT mice. Sprague Dawley pregnant rats were ordered from B&K Universal Ltd, Sollentuna, Sweden (hppt://www.bku.com). All animals were housed in groups with ad libitum access to food and water at a 12-h light/dark cycle. All experimental procedures conducted in this study had been approved by the Ethical Committee at Lund University.

For immunohistochemical analysis, mice (from two weeks old and adult stage) and juvenile rats were sacrificed by transcardial perfusion with saline for 5–10 minutes, followed by 4% paraformaldehyde (PFA) for 10 minutes. Brains were kept in PFA overnight at 4°C and subsequently cryopreserved in a 20–30% sucrose/0.1 M phosphate buffer solution until sectioning on a microtome apparatus (30 µm thickness sections, Microm Zeiss). Seven series of coronal sections were cut throughout the brain. Free-floating sections were preserved in antifreeze solution until immunohistochemistry was performed. Heads of postanatal two days old mice were decapitated and soaked in PFA 4% for 24 hours, at 4°C and transferred into sucrose solution until sectioning on cryostat apparatus (16 µm thickness; Leica CM3000). Sections were mounted on Superfrost glass slides and stored at −80°C until immunohistochemistry was performed.

### Cloning, subcloning, virus production and titer measurement

The Moloney leukemia-derived retroviral vectors used in this study, pCMMP-IRES2eGFP-WPRE and pCMMP-Ngn2-IRES2eGFP-WPRE were previously described [40], [49]. To generate the construct pCMMP-NeuroD1-IRES2eGFP-WPRE, mouse NeuroD1 cDNA was amplified from a pCS2+mtNeuroD1 plasmid (kindly provided by Professor Jackie Lee, Denver university, Boulder, USA) by PCR to introduce the restriction sites PmeI in 5′ and XhoI in 3′. Amplification of cDNA was performed as previously described [49]. The construction was verified by enzymatic restriction and by DNA sequencing using BigDye 3.1 (ABI). All infectious particles were produced using the producer cell line 293VSV-G and as previously described [49]. The titer of each retrovirus was measured by flow-cytometry based on eGFP expression, four days following infection of HT1080 cells and ranged from 0.5×10^9^−2.1×10^9^ TU/ml (All details on how to produce infectious particles can be provided upon request).

### Neurosphere generation, transduction and differentiation

Pregnant female Sprague-Dawley rats (B & K Universal, Sollentuna, Sweden) were terminally anesthetized by an overdose of sodium pentobarbital (i.p., 60 ng/ml). Embryos at stage embryonic day E14.5 (Plug day as day 0) were collected and cortical-hippocampal neurospheres were generated following dissection of the dorso-posterior part of the cortical tissue, and generated as previously described [49]. In this study, second passage (P2) neurospheres were used to study the effect of the overexpression of Ngn2 and NeuroD1 on neuronal differentiation. Each well, containing an equal starting population of 200,000 cells/ml, corresponding to 15–25 neurospheres, was transduced independently with each retrovirus at a multiplicity of infection (MOI) of 1, in proliferation medium supplemented by protamine-sulfate (4 mg/ml, Sigma). To induce differentiation, the medium was replaced with normal basic differentiation medium two days post-transduction and subsequently changed every other day until fixation.

### Immunocytochemistry, immunohistochemistry and microscopy

The antibodies used in this study are: rabbit anti-GFAP (1∶1000; DAKO), mouse anti-Nestin (1∶100; BD PharMingen), guinea pig anti-Glast (1∶500; Chemicon), rabbit anti-Prox1 (1∶1000; Covance), goat anti-Ngn2 (1∶20; Santa Cruz), goat anti-NeuroD1 (1∶200; Santa Cruz), rabbit anti-Pax6 (1∶150; Covance), rabbit anti-Trb2 (1∶500; Chemicon), rabbit anti-Trb1: (1∶1000; Chemicon), goat anti-Dcx: (1∶500; Santa Cruz), mouse anti-PSA-NCAM: (1∶500; Chemicon), mouse anti-NeuN (1∶300; Chemicon), rabbit anti-Calretinin (1∶500; Swant); mouse anti-Sox2 (1∶100; R&D systems), mouse anti-Mash1 (1∶100; BD PharMingen), rabbit anti-NeuroD2 (1∶300; ABCAM), mouse anti-MAP2 (1∶500; Sigma), rabbit anti-Ki67 (1∶150; NovaCastra) and rabbit anti-phospho-Histone H3. The secondary antibodies (1∶200) Cy2, FITC, Cy3 and Cy5 were from Jackson IR laboratories, Alexa-fluor 488, 568, 595 and 647 from Invitrogen-Molecular Probes. DAPI (1∶1000) was purchased from Sigma.

For immunocytochemistry, cultures were fixed in 4% paraformaldehyde at day five, rinsed with PBS three times prior to pre-incubation with a blocking solution (10% donkey serum, 0.25% TritonX100 in PBS) for 1 hour. The remainder of the procedure was performed as previously described [49]. Specimen analyses were performed using a Leica confocal microscope (Leica software, equipped with a GreNe and a HeNe laser, using the following lines of excitation: 488 nm, 594 nm and 647 nm). Samples were analyzed using 20×, 40× and 63× objectives, sometimes zoomed. Figures were composed in CANVAS-X software.

### Bromo-deoxyuridine (BrdU) and Cloro-deoxyuridine (CldU) pulse labeling and immunohisto- and immunocyto-chemistry

To assess cell proliferation and ongoing neurogenesis in vivo, animals were injected with BrdU (100 mg/kg, Sigma), two hours prior to sacrifice (for both P2 and two weeks old Ngn2 mice). To assess neurogenesis, BrdU (100 mg/kg) was injected 48 hours prior to sacrifice (for P2 animals, BrdU was injected in pregnant dams half day prior to give birth). Immunohistochemistry was carried on as described above using a rat anti-CldU/BrdU primary antibody (1∶200, monoclonal, Immunologicalsdirect, Oxfordshire, UK), with an additional denaturation in 1 M HCl for 30 minutes prior to pre-incubation with serum.

To assess the neuronal-inducing activity of Ngn2 and NeuroD1, transduced cultures were incubated with CldU (20 µM, Sigma) for 2 days, after a period of differentiation of five days. For immunocytochemistry, cultures were fixed with 4% paraformaldehyde at day 7, rinsed with PBS three times, treated with 1 M HCl at 65°C for 5–10 min, pre-incubated and then incubated with a rat anti-CldU/BrdU antibody and other primary antibodies. The remainder of the procedure was performed according to the protocol for immunohistochemistry already mentioned.

### In utero surgery

Timed pregnant female Sprague Dawley rats with embryos at gestational age E15.5 were anesthetized with halothane. The mother was placed in the lower level of a two-level wooden stage. The abdomen was shaved with an electric razor and then cleaned with 70% alcohol. A 2–3 cm midline laparotomy was performed. Each uterine horn was carefully taken out individually and the number of embryos recorded. One horn was then placed back inside the mother, whilst the other horn was prepared for injection. The embryos were kept moist with constant application of warm saline to prevent dehydration. Approximately 2 µl of viral suspension (1×10^∧^9TU/ml) was injected into the lateral ventricle of each embryo, except for the embryo closest to the vagina. After the injections, the uterine horns were placed back into the abdomen. The abdominal wall and the overlying skin were then sutured. Care was taken not to damage abdominal muscles so that normal delivery of the pups was possible at term. The entire surgery generally took about 45 minutes to one hour. Each mother was allowed to recover in her cage before being returned to the animal stable. Shredded paper was added to each cage to encourage nesting and special care was taken not to stress the mothers. Following normal delivery, the pups were allowed to develop to adulthood up to two weeks.

### Quantification

For the in vivo experiment, manual cell counting was performed on 18 and 30 µm thick brain sections for two days and two weeks old animals, respectively. The brain of P2 animals was cut into 10 series of coronal sections; the brain of two-weeks old animals was cut into 7–8 series of coronal sections. For each staining, one-two series from three to five different individuals were analyzed per genotype using a confocal microscope with 1, 2 or 3 lines of excitation, in sequential scanning in order to avoid false positives. When two series were analyzed, only one series was counted. The mean number of cells per hippocampus expressing the markers of interest, was calculated for each brain, based on the analysis of 6–8 consecutive dorsal hippocampal sections. The final mean number of cells per section was calculated by adding the mean number from each individual. We expressed the data as the mean number of positive cells±standard error of the mean (SEM). In this study, more than 25 Ngn2 null, >45 Ngn2 heterozygotes, and >60 WT individuals (including rats injected with retroviruses) were analyzed, in total. Statistical comparison was performed using one-factor analysis of variance (ANOVA) with transcription factor, cellular marker, genotype or time as variables, followed by post-hoc analysis when significant differences were observed, using Statview 5.0 software (SAS institute Inc.). For cell culture experiments, three independent experiments were performed, each in duplicate. The counting was based on seven randomly chosen different fields of view. For each diagram, the level of significance (p-value) is represented as follows: P<0.05 = *; P<0.001 = **; P<0.0001 = ***. All data are expressed as±standard error of the mean (SEM).

### Human sample and RT-PCR

The brain from a 64 year-old male was provided by the Harvard Brain Tissue Resource Center. The individual whose brain tissue was being analyzed gave written consent for storage and use of his tissue for research. Five millimeters thick fresh human hippocampal tissue sample was snap frozen. The frozen tissue block was cut into 20 series of coronal sections, using a cryostat. Every 10 sections, one section was placed in an Eppendorf tube, on dry ice. Pooled sections were used for RT-PCR. Total RNA was prepared using Trizol and RNAeasy, supplemented with RNAGuard as previously described [49]. The RNA was digested with shrimp DNAse before cDNA synthesis, which was performed using a mix of oligo-dT and random hexamer primers and SuperscriptII reverse transcriptase. Advantage2 polymerase mix (Clontech/BRL) was used for PCR, with the following cycling conditions: 10 cycles of 94° C for 30 seconds, 68°C for 2 minutes, 30 cycles of 94°C for 30 seconds, 60°C for 1 minute, 68°C for 1 minute and 30 seconds, one cycle of 68° C for 2 minutes, soak at 16°C. Primers marked * were designed by PrimerBank. hRPS18 (sense) 5′-GCCTTTGCCATCACTGCCATT and hRPS18 (antisense) 5′-GCCAGTGGTCTTGGTGTGCT, hPAX6 (sense) 5′-GCCCTGGAGAAAGAGTTTGAGAGAACCCATT hPAX6 (antisense) 5′-GGGGAAATGAGTCCTGTTGAAGTGGTGC, hTBR1 (sense) 5′-GCGGACACCAATGTGCAAGGAAATCG and hTBR1 (antisense) 5′-CGAGGGGGTCAGGCGGTCCATGTCACAGC, hTBR2 (sense) 5′-GACCTGTGGCAAAGCCGACAATAACATGC and hTBR2 (antisense) 5′-GGGGGTGTCTCTATCCAAGAAGAGCCAAT, hPDHX ( = PROX1) (sense) 5′-GGGACACTACGGTTCCGTTTAAGTCCAGC and hPDHX (antisense) 5′-CTCTCCATCCCAGCTTACATTAACATCTGGCATTTGT, hNGN2 (sense) * 5′-CATCAAGAAGACCCGTAGACTGA and hNGN2 (antisense)* 5′-CAACACTGCCTCGGAGAAG, hMASH1 (sense) 5′-CCTGGATCCGCATGGAAAGCTCTGCCAAGATGGAG and hMASH1 (antisense) 5′-CCTGGATCCCCCCTCAGAACCAGTTGGTGAAGTCGA, hNEUROD1 (sense) 5′-GCTCAGGACCTACTAACAACAAAGGAAATCGAAACATG and hNEUROD1 (antisense) 5′-CAAAGCGTCTGAACGAAGGAGACCAGGT, hGLAST (sense) 5′-CATCAGGGAAGATGGGAATGCGAGCTGTAGTCTATTAT and hGLAST (antisense) 5′-CCACGGGGGCATACCACATTATTACTGCTACC, hNEST (sense) 5′-CAGGAGCGGCTGCGGGCTACTGAAAAGTTCC and hNEST (antisense) 5′-CAGGGCTGAGGGGTGGTGCCAAGGAGG, hGFAP (sense) 5′-CCACGAGGAGGAGGTTCGGGAACTCCAGGAGC and hGFAP (antisense) 5-GGAATGGTGATCCGGTTCTCCTCGCCCTCTAGC, hCALB2 ( = Calretinin) (sense) 5′-GGCTCTGGCATGATGTCAAAGAGTGACAACTTT and hCALB2 (antisense) 5′-GGGCATCCAGCTCATGCTCGTCAATGTAGCC, hCALB1 (sense) 5′-GCGAAAGAAGGCTGGATTGGAGTTATCACC and hCALB1 (antisense) 5′-CCCTCCATCCGACAAAGCCATTATGTTCTTCTTGTATG, hSOX2 (sense) 5′-GGAGAACCCCAAGATGCACAACTCGGAGAT and hSOX2 (antisense) 5′-GAGGAAGAGGTAACCACAGGGGGGCTGGAGC, hWNT3A (sense) 5′-CCGAGGGCATCAAGATTGGCATCCAGGAGTGC and hWNT3A (antisense) 5′-TCGGGTTGCGACCACCAGCATGTCTTCACCTC, hSHH* (sense) 5′-ACTCCGAGCGATTTAAGGAACT and hSHH* (antisense) 5′-CAGACGTGGTGATGTCCACTG.

## Supporting Information

Figure S1 Characterization of the molecular signatures defining each phases of cell maturation during hippocampal granule neuronal differentiation on coronal sections from two months old mice. (A1–I2) Indirect immunofluorescence performed on hippocampal coronal sections from two months old WT mice. (A1–B2) Type-1 radial glia stem cells are identified by the co-expression of Nestin, Glast, GFAP and Pax6 and their characteristic baso-apical orientation. Radial glia cells are located in both dorsal and ventral blades of the DG. (C1–C2) Type-2 amplifying progenitors expressing Tbr2 rarely co-express Dcx. (D1–D2) Type-3 maturing granule neurons co-express Tbr1, PSA-NCAM and Dcx. Tbr1 expression is weakly maintained in mature hippocampal granule neurons. (E1–E2) Type-3 maturing granule neurons co-express both NeuroD1 and PSA-NCAM but not Pax6. (F1–F2) Granule neurons maturation occurs through the expression of NeuroD1, PSA-NCAM and Calretinin. (G1–G2) The transition from type-2 amplifying progenitors to type-3 immature neurons occurs at the onset of Tbr2 expression and the beginning of that of NeuroD1. (H1–H2) The transition from type-3 amplifying progenitors to mature granule neurons occurs through the sequential expression of NeuroD1, Tbr1 and NeuN. (I1–I2) The expression of Prox1 starts just after that of NeuroD1. (J) In the DG of two weeks old newborn mice, more than 80% of Mash1-positive cells were found co-expressing Ki67. Panels A2–I2 represent a high magnification of framed areas in corresponding panels A1–I1. Rectangular images on the bottom and right of the panels A2–I2 represent projected images of 14-Z stacks (total of 10–14 µm thick). The white crosshairs in these panels were positioned to show single cells co-expressing markers of interest, as labeled above each panel. Scale bars: 50 µm (A1, B1, C1, D1, E1, F1, G1, H1 and I1), 25 µm (A2, B2, C2, D2, E2, F2, G2, H2, I2 and J).
(8.74 MB TIF)

Figure S2 The absence of Ngn2 induces an up-regulation of NeuroD1 in neuronal progenitors and an impaired production of GFAP astrocytes. (A1–F2) Indirect immunofluorescence performed on hippocampal coronal sections from two days old Ngn2+/GFP and Ngn2GFP/GFP mutant animals. (A1–B2) In absence of Ngn2 protein, a uniform reduced size of the hippocampus, including the DG, is observed through the rostro-caudal axis. The up regulation of NeuroD1 in migrating neuroblasts and the presence of the marker in the CA3 region in Ngn2GFP/GFP animals suggest a delay in neuroblasts production in absence of Ngn2. (C–D4′) Pax6-positive migrating cells migrates towards the developing DG can be observed in the hippocampus of both Ngn2+/GFP and Ngn2GFP/GFP animals. (E1–G) The absence of Ngn2 has an impact of GFAP astrocytes production. The scaffold made by migrating GFAP astrocytes is partially absent in Ngn2GFP/GFP animals and might be the result of the mal-positioning of some of the GFP+ cells we observed located outside of the granule layer in the DG of two weeks old Ngn2GFP/GFP mutant animals (See Figure 4L1 and supplementary Figure 3A1). Panels C* and D4′* represent a high magnification of framed areas in corresponding panels C and D4′. Rectangular images on the bottom and right of these panels represent projected images of 10-Z stacks (total of 10–14 µm thick). The white crosshairs in these panels were positioned to show single cells co-expressing markers of interest, as labeled above each panel. In E2, the dashed and plain lines delineate the different population of GFAP astrocytes (red), Ngn2 migrating progenies (green) and NeuroD1 maturing neuroblasts (blue). SVZ = subventricular zone, hSVZ = hippocampal subventricular zone, CA = cornus ammonis, RG = radial glia, AP = astrocyte precursor and NP = neuronal progenitor. Scale bars: 200 µm (A1–B2, E1 and F1), 50 µm (C–D4, D1–D4′, E2 and F2), 25 µm (D4*, D4′* and C*).
(4.24 MB TIF)

Figure S3 Granule neuron identity is conserved in absence of Ngn2 protein. (A1–G2) Indirect immunofluorescence performed on hippocampal coronal sections of two weeks old Ngn2GFP/GFP mutant animals and two days old Ngn2+/GFP and Ngn2GFP/GFP mutant animals. (A1–A4) In two weeks old Ngn2GFP/GFP mutant animals, Pax6-positive stem cells give rise to GFPbright-expressing cells, which undergo granule neuron maturation as marked by the co-expression of GFPlow and NeuroD1. Arrowheads pinpoint at mis-located GFP cells. (B1 and B2) In absence of Ngn2, Tbr2 expression is not affected in granule neuron progenitors. (C1 and C2) The presence of Mash1-positive cells is not altered in Ngn2GFP/GFP mutant animals. Mash1-positive cells give rise to GFP-expressing progenies. (D1–G4) In both presence and absence of Ngn2 protein in two days old mice, hippocampal granule neuron differentiation occurs and granule identity is conserved, as marked by the expression of Tbr1 and Prox1. Panels A4, B2, C2, D2, E2, F2 and G2 represent a high magnification of framed areas in corresponding panels A3, B1, C1, D1, E1, F1 and G1. Rectangular images on the bottom and right of these panels represent projected images of 14-Z stacks (total of 10–14 µm thick). The white crosshairs in these panels were positioned to show co-expression in single cells of markers of interest, as labeled above each panel. Scale bars: 100 µm (B1, F1 and J1), 50 µm (A1–3, C1, D1 and E1), 25 µm (A4, B2, C2, D2, F2 and G2), 5 µm (E2).
(6.39 MB TIF)

Figure S4 The symmetric division of granule neuroblasts coincides with NeuroD1 expression (A1–E2) Indirect immunofluorescence performed on hippocampal coronal sections of two weeks old Ngn2+/GFP and Ngn2GFP/GFP mutant animals and two weeks and two months old WT mice. (A1–A9) In Ngn2+/GFP mice, GFP-expressing cells undergo symmetric division and give rise to GFP/NeuroD1-positive cells. In these cells, a haze-like and punctuate pattern of distribution of NeuroD1 protein was observed. The pattern of NeuroD1 in these cells is characteristic of newly processed mRNA located in structures known as speckles or interchromatin granule clusters (Zeng et al., 1997). (B1–B2) Another example of a NeuroD1-positive cell which divides symmetrically. (C1 and C2) In Ngn2GFP/GFP mutant animals, only a few GFP/PH3-positive NeuroD1-negative cells divide. (D1–E2) Ki67, marker of end phases of the cell cycle, is expressed in NeuroD1-positive PSA-NCAM-negative cells in the DG SGZ of two weeks and two months old WT mice. Rectangular images on the bottom and right of the panels A5–A9, B2, C2, D2 and E2 represent a projection of 14-Z stacks images (total of 10–14 µm thick) from framed areas or pointing arrows in panels A1–A4, B1, C1, D1 and E1, respectively. The white crosshairs in these panels were positioned to show co-expression in single cells of markers of interest, as labeled above each panel. Arrows and arrowheads point at cells of interest. Scale bars: 50 µm (A1–4, B1, C1, D1 and E1), 25 µm (B2, C2, D2 and E2), 5 µm (A5–8).
(4.15 MB TIF)

Figure S5 Ngn2 and NeuroD1 induce neuronal differentiation of E14.5 cortico-hippocampal neuropsheres. (A1–E4) Indirect immunofluorescence performed on E14.5 WT cortico-hippocampal neuropsheres. (A1–C4) Ngn2- and NeuroD1-transduced neural progenitors differentiated for five days in vitro downregulate Pax6 and mature into MAP2-positive neurons. (D1-E4) Ngn2-transduced cells strongly express Pax6 when dividing. Panels D4 and E4 represent a high magnification of framed areas in panels D2 and E2, respectively. Scale bars: 50 µm (A1–D3 and E1–3).
(4.26 MB TIF)

Figure S6 NeuroD1 overexpression induces neuronal granule neuron maturation in vivo. (A–B6) Indirect immunofluorescence performed on hippocampal coronal sections of two weeks old rats that were injected with NeuroD1 retrovirus at the embryonic age of E15.5. (A) The overexpression of NeuroD1 results in the generation of hippocampal DG granule neurons. These neurons (NeuroD1/GFP -positive) express the mature neuronal marker NeuN and are very often localized in the external granule layer (first layers generated during the DG development). (B1–B6) A few cells transduced with NeuroD1 retrovirus (GFP-positive) have a glial-like shape. These cells still express NeuroD1. Rectangular images on the bottom and right of the panels B4–B6 represent a projection of 10-Z stacks images (total of 10–14 µm thick) from framed areas in panels B1 The white crosshairs in these panels were positioned to show co-expression in single cells of markers of interest, as labeled above each panel. Scale bars: 50 µm (A–B3), 10 µm (B4–6).
(4.44 MB TIF)
