# Supplementary figures and images for: Neurogenin2 Directs Granule Neuroblast Production and Amplification while NeuroD1 Specifies Neuronal Fate during Hippocampal Neurogenesis
Source: PLoS One. 2009 Mar 10;4(3):e4779. doi: 10.1371/journal.pone.0004779 (PMC2652712; doi:10.1371/journal.pone.0004779)

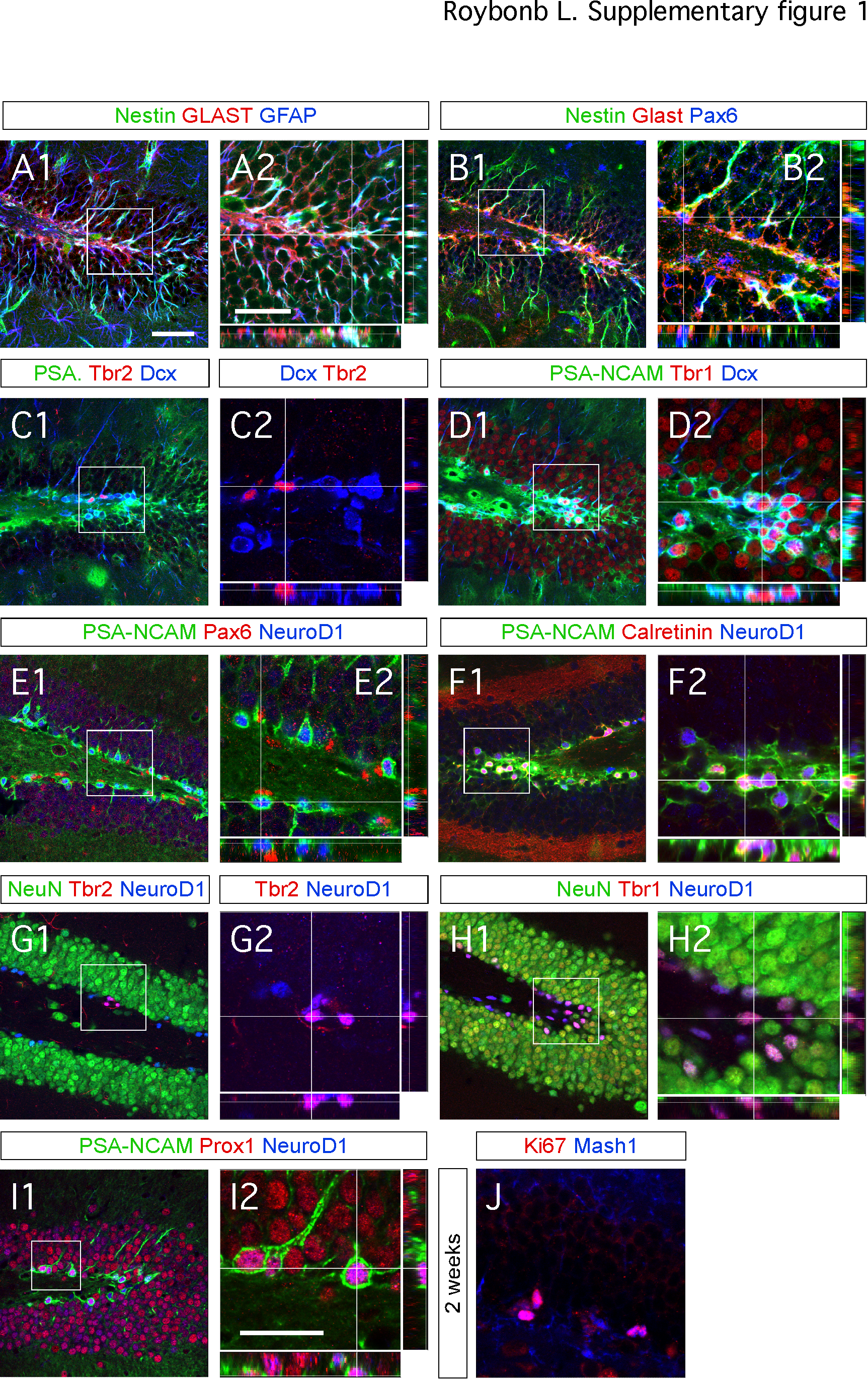

Supplement: Figure S1 — Characterization of the molecular signatures defining each phases of cell maturation during hippocampal granule neuronal differentiation on coronal sections from two months old mice. (A1–I2) Indirect immunofluorescence performed on hippocampal coronal sections from two months old WT mice. (A1–B2) Type-1 radial glia stem cells are identified by the co-expression of Nestin, Glast, GFAP and Pax6 and their characteristic baso-apical orientation. Radial glia cells are located in both dorsal and ventral blades of the DG. (C1–C2) Type-2 amplifying progenitors expressing Tbr2 rarely co-express Dcx. (D1–D2) Type-3 maturing granule neurons co-express Tbr1, PSA-NCAM and Dcx. Tbr1 expression is weakly maintained in mature hippocampal granule neurons. (E1–E2) Type-3 maturing granule neurons co-express both NeuroD1 and PSA-NCAM but not Pax6. (F1–F2) Granule neurons maturation occurs through the expression of NeuroD1, PSA-NCAM and Calretinin. (G1–G2) The transition from type-2 amplifying progenitors to type-3 immature neurons occurs at the onset of Tbr2 expression and the beginning of that of NeuroD1. (H1–H2) The transition from type-3 amplifying progenitors to mature granule neurons occurs through the sequential expression of NeuroD1, Tbr1 and NeuN. (I1–I2) The expression of Prox1 starts just after that of NeuroD1. (J) In the DG of two weeks old newborn mice, more than 80% of Mash1-positive cells were found co-expressing Ki67. Panels A2–I2 represent a high magnification of framed areas in corresponding panels A1–I1. Rectangular images on the bottom and right of the panels A2–I2 represent projected images of 14-Z stacks (total of 10–14 µm thick). The white crosshairs in these panels were positioned to show single cells co-expressing markers of interest, as labeled above each panel. Scale bars: 50 µm (A1, B1, C1, D1, E1, F1, G1, H1 and I1), 25 µm (A2, B2, C2, D2, E2, F2, G2, H2, I2 and J). (8.74 MB TIF) [file pone.0004779.s001.tif]

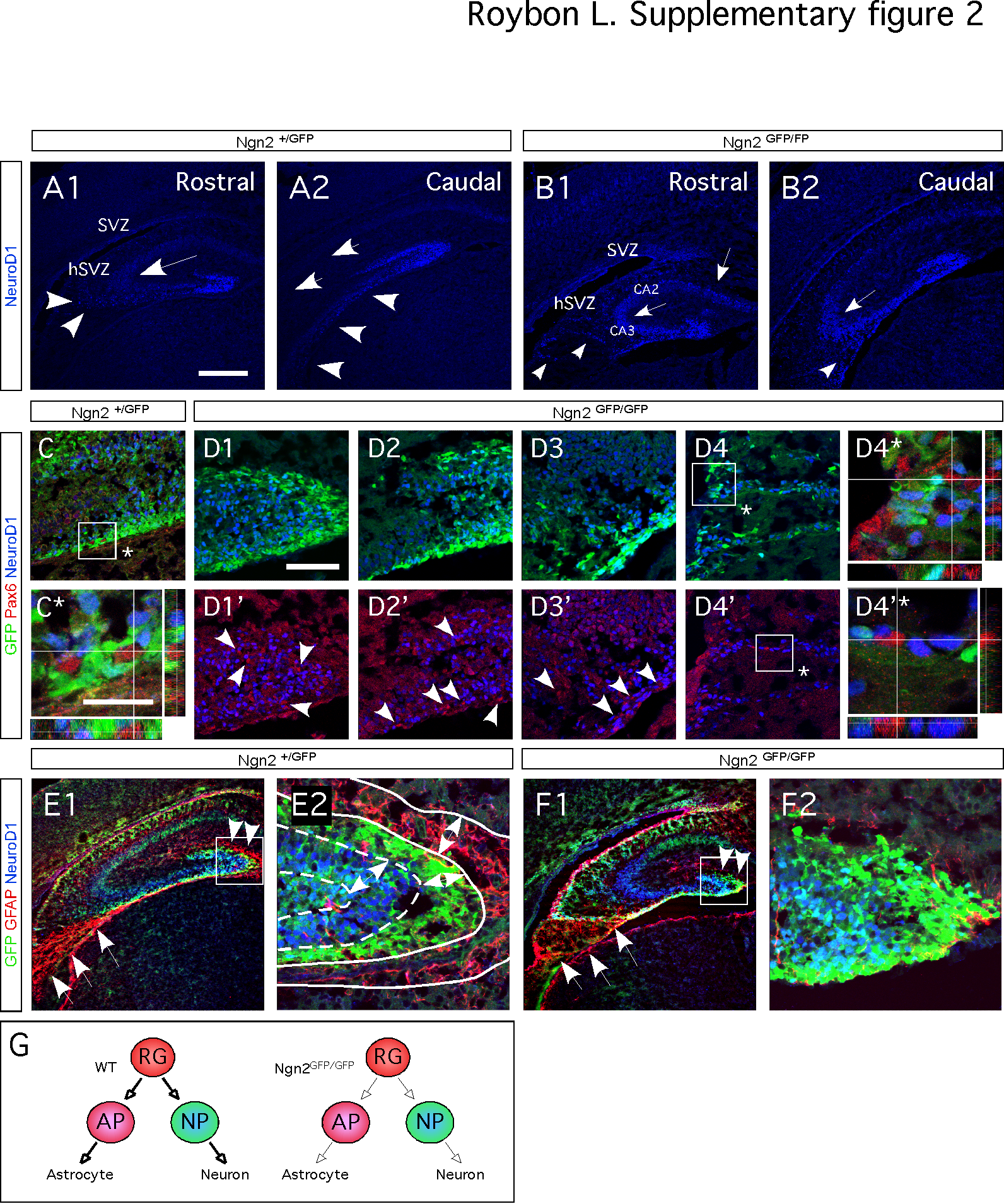

Supplement: Figure S2 — The absence of Ngn2 induces an up-regulation of NeuroD1 in neuronal progenitors and an impaired production of GFAP astrocytes. (A1–F2) Indirect immunofluorescence performed on hippocampal coronal sections from two days old Ngn2+/GFP and Ngn2GFP/GFP mutant animals. (A1–B2) In absence of Ngn2 protein, a uniform reduced size of the hippocampus, including the DG, is observed through the rostro-caudal axis. The up regulation of NeuroD1 in migrating neuroblasts and the presence of the marker in the CA3 region in Ngn2GFP/GFP animals suggest a delay in neuroblasts production in absence of Ngn2. (C–D4′) Pax6-positive migrating cells migrates towards the developing DG can be observed in the hippocampus of both Ngn2+/GFP and Ngn2GFP/GFP animals. (E1–G) The absence of Ngn2 has an impact of GFAP astrocytes production. The scaffold made by migrating GFAP astrocytes is partially absent in Ngn2GFP/GFP animals and might be the result of the mal-positioning of some of the GFP+ cells we observed located outside of the granule layer in the DG of two weeks old Ngn2GFP/GFP mutant animals (See Figure 4L1 and supplementary Figure 3A1). Panels C* and D4′* represent a high magnification of framed areas in corresponding panels C and D4′. Rectangular images on the bottom and right of these panels represent projected images of 10-Z stacks (total of 10–14 µm thick). The white crosshairs in these panels were positioned to show single cells co-expressing markers of interest, as labeled above each panel. In E2, the dashed and plain lines delineate the different population of GFAP astrocytes (red), Ngn2 migrating progenies (green) and NeuroD1 maturing neuroblasts (blue). SVZ = subventricular zone, hSVZ = hippocampal subventricular zone, CA = cornus ammonis, RG = radial glia, AP = astrocyte precursor and NP = neuronal progenitor. Scale bars: 200 µm (A1–B2, E1 and F1), 50 µm (C–D4, D1–D4′, E2 and F2), 25 µm (D4*, D4′* and C*). (4.24 MB TIF) [file pone.0004779.s002.tif]

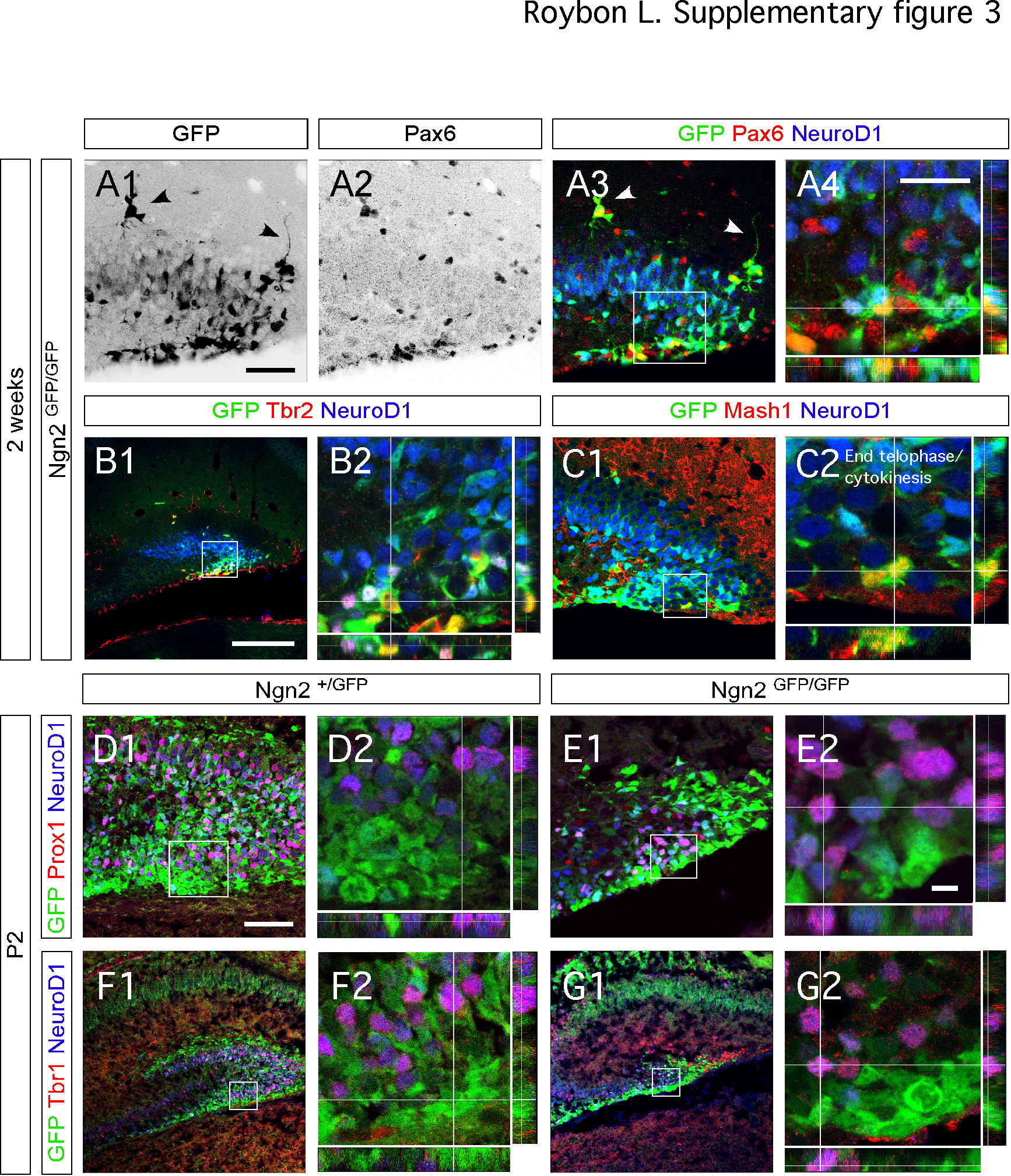

Supplement: Figure S3 — Granule neuron identity is conserved in absence of Ngn2 protein. (A1–G2) Indirect immunofluorescence performed on hippocampal coronal sections of two weeks old Ngn2GFP/GFP mutant animals and two days old Ngn2+/GFP and Ngn2GFP/GFP mutant animals. (A1–A4) In two weeks old Ngn2GFP/GFP mutant animals, Pax6-positive stem cells give rise to GFPbright-expressing cells, which undergo granule neuron maturation as marked by the co-expression of GFPlow and NeuroD1. Arrowheads pinpoint at mis-located GFP cells. (B1 and B2) In absence of Ngn2, Tbr2 expression is not affected in granule neuron progenitors. (C1 and C2) The presence of Mash1-positive cells is not altered in Ngn2GFP/GFP mutant animals. Mash1-positive cells give rise to GFP-expressing progenies. (D1–G4) In both presence and absence of Ngn2 protein in two days old mice, hippocampal granule neuron differentiation occurs and granule identity is conserved, as marked by the expression of Tbr1 and Prox1. Panels A4, B2, C2, D2, E2, F2 and G2 represent a high magnification of framed areas in corresponding panels A3, B1, C1, D1, E1, F1 and G1. Rectangular images on the bottom and right of these panels represent projected images of 14-Z stacks (total of 10–14 µm thick). The white crosshairs in these panels were positioned to show co-expression in single cells of markers of interest, as labeled above each panel. Scale bars: 100 µm (B1, F1 and J1), 50 µm (A1–3, C1, D1 and E1), 25 µm (A4, B2, C2, D2, F2 and G2), 5 µm (E2). (6.39 MB TIF) [file pone.0004779.s003.tif]

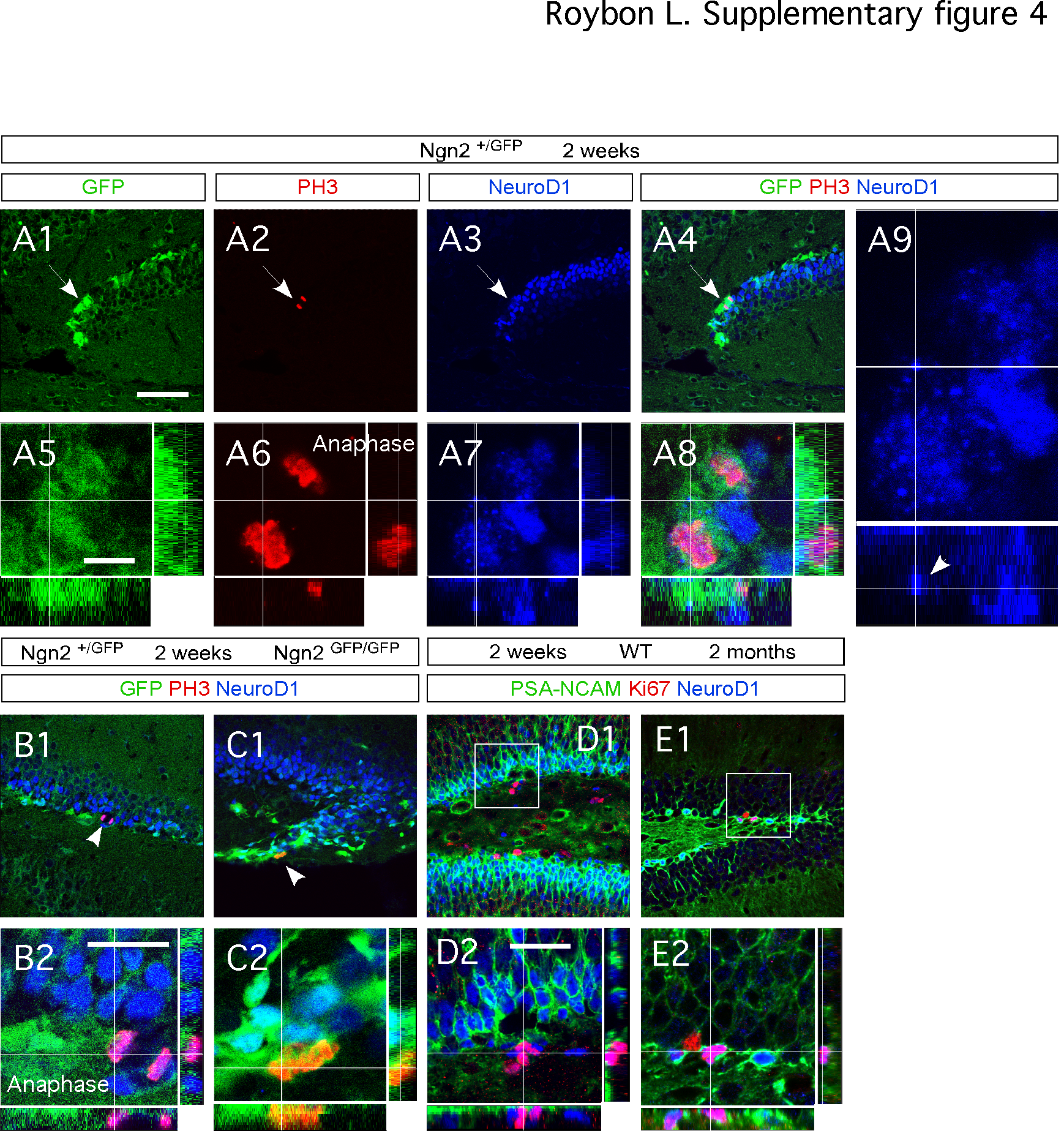

Supplement: Figure S4 — The symmetric division of granule neuroblasts coincides with NeuroD1 expression (A1–E2) Indirect immunofluorescence performed on hippocampal coronal sections of two weeks old Ngn2+/GFP and Ngn2GFP/GFP mutant animals and two weeks and two months old WT mice. (A1–A9) In Ngn2+/GFP mice, GFP-expressing cells undergo symmetric division and give rise to GFP/NeuroD1-positive cells. In these cells, a haze-like and punctuate pattern of distribution of NeuroD1 protein was observed. The pattern of NeuroD1 in these cells is characteristic of newly processed mRNA located in structures known as speckles or interchromatin granule clusters (Zeng et al., 1997). (B1–B2) Another example of a NeuroD1-positive cell which divides symmetrically. (C1 and C2) In Ngn2GFP/GFP mutant animals, only a few GFP/PH3-positive NeuroD1-negative cells divide. (D1–E2) Ki67, marker of end phases of the cell cycle, is expressed in NeuroD1-positive PSA-NCAM-negative cells in the DG SGZ of two weeks and two months old WT mice. Rectangular images on the bottom and right of the panels A5–A9, B2, C2, D2 and E2 represent a projection of 14-Z stacks images (total of 10–14 µm thick) from framed areas or pointing arrows in panels A1–A4, B1, C1, D1 and E1, respectively. The white crosshairs in these panels were positioned to show co-expression in single cells of markers of interest, as labeled above each panel. Arrows and arrowheads point at cells of interest. Scale bars: 50 µm (A1–4, B1, C1, D1 and E1), 25 µm (B2, C2, D2 and E2), 5 µm (A5–8). (4.15 MB TIF) [file pone.0004779.s004.tif]

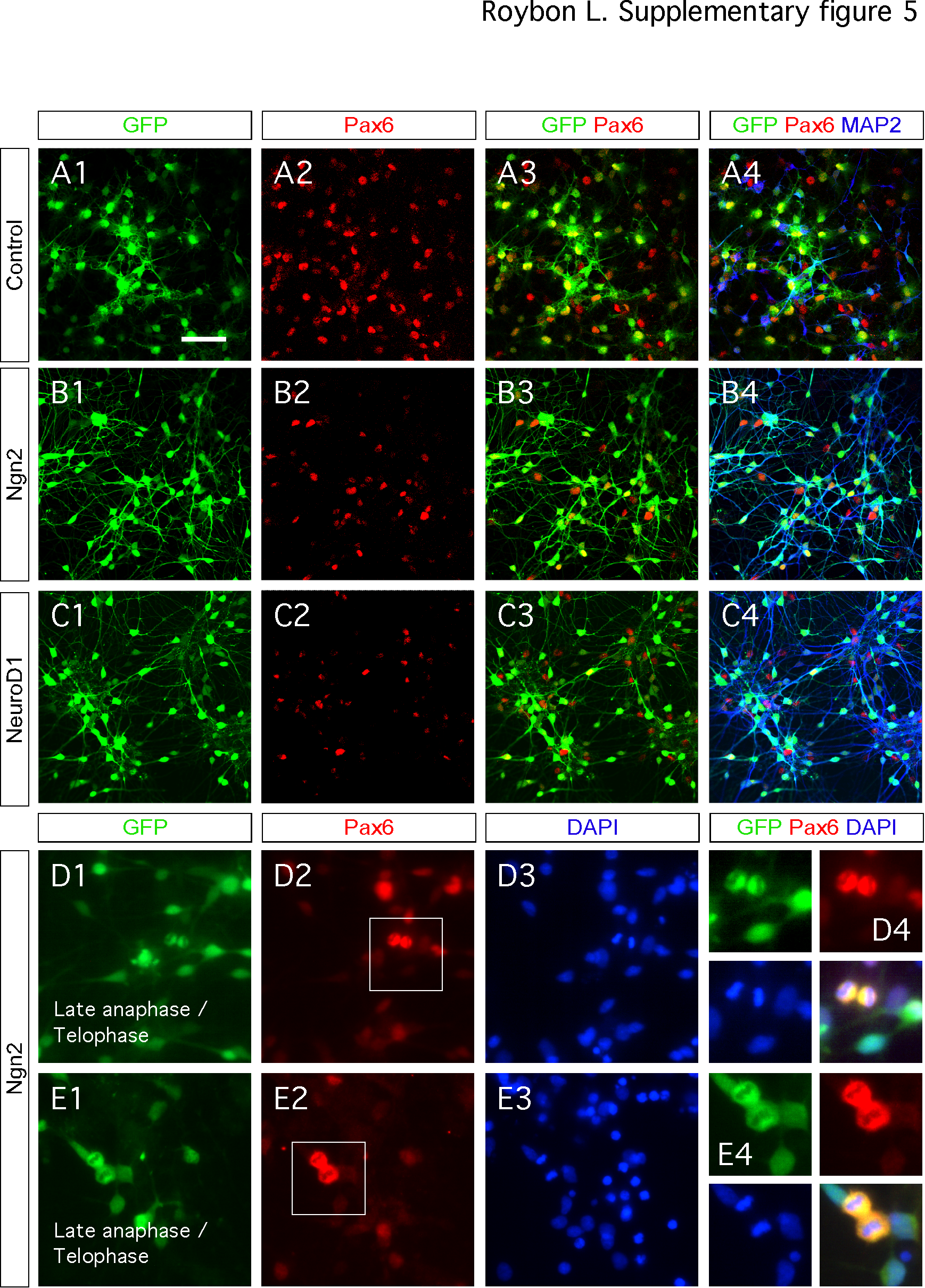

Supplement: Figure S5 — Ngn2 and NeuroD1 induce neuronal differentiation of E14.5 cortico-hippocampal neuropsheres. (A1–E4) Indirect immunofluorescence performed on E14.5 WT cortico-hippocampal neuropsheres. (A1–C4) Ngn2- and NeuroD1-transduced neural progenitors differentiated for five days in vitro downregulate Pax6 and mature into MAP2-positive neurons. (D1-E4) Ngn2-transduced cells strongly express Pax6 when dividing. Panels D4 and E4 represent a high magnification of framed areas in panels D2 and E2, respectively. Scale bars: 50 µm (A1–D3 and E1–3). (4.26 MB TIF) [file pone.0004779.s005.tif]

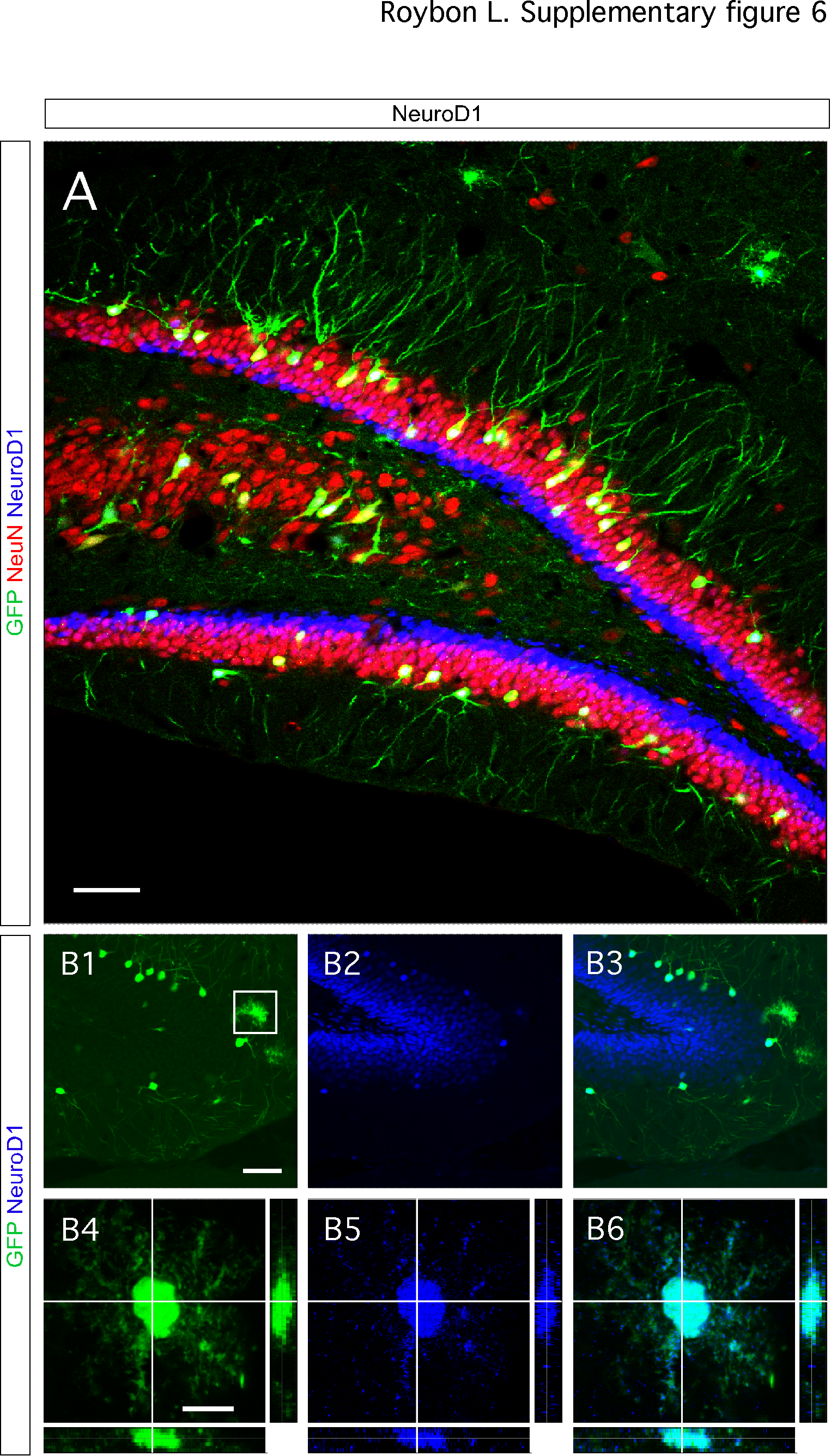

Supplement: Figure S6 — NeuroD1 overexpression induces neuronal granule neuron maturation in vivo. (A–B6) Indirect immunofluorescence performed on hippocampal coronal sections of two weeks old rats that were injected with NeuroD1 retrovirus at the embryonic age of E15.5. (A) The overexpression of NeuroD1 results in the generation of hippocampal DG granule neurons. These neurons (NeuroD1/GFP -positive) express the mature neuronal marker NeuN and are very often localized in the external granule layer (first layers generated during the DG development). (B1–B6) A few cells transduced with NeuroD1 retrovirus (GFP-positive) have a glial-like shape. These cells still express NeuroD1. Rectangular images on the bottom and right of the panels B4–B6 represent a projection of 10-Z stacks images (total of 10–14 µm thick) from framed areas in panels B1 The white crosshairs in these panels were positioned to show co-expression in single cells of markers of interest, as labeled above each panel. Scale bars: 50 µm (A–B3), 10 µm (B4–6). (4.44 MB TIF) [file pone.0004779.s006.tif]
